# Possibilistic distribution distance metric: a robust domain adaptation learning method

**DOI:** 10.3389/fnins.2023.1247082

**Published:** 2023-11-09

**Authors:** Jianwen Tao, Yufang Dan, Di Zhou

**Affiliations:** ^1^Institute of Artificial Intelligence Application, Ningbo Polytechnic, Zhejiang, China; ^2^Industrial Technological Institute of Intelligent Manufacturing, Sichuan University of Arts and Science, Dazhou, China

**Keywords:** electroencephalogram, domain adaptation, probabilistic clustering, maximum mean discrepancy, fuzzy entropy

## Abstract

The affective Brain-Computer Interface (aBCI) systems, which achieve predictions for individual subjects through training on multiple subjects, often cannot achieve satisfactory results due to the differences in Electroencephalogram (EEG) patterns between subjects. One tried to use Subject-specific classifiers, but there was a lack of sufficient labeled data. To solve this problem, Domain Adaptation (DA) has recently received widespread attention in the field of EEG-based emotion recognition. Domain adaptation (DA) learning aims to solve the problem of inconsistent distributions between training and test datasets and has received extensive attention. Most existing methods use Maximum Mean Discrepancy (MMD) or its variants to minimize the problem of domain distribution inconsistency. However, noisy data in the domain can lead to significant drift in domain means, which can affect the adaptability performance of learning methods based on MMD and its variants to some extent. Therefore, we propose a robust domain adaptation learning method with possibilistic distribution distance measure. Firstly, the traditional MMD criterion is transformed into a novel possibilistic clustering model to weaken the influence of noisy data, thereby constructing a robust possibilistic distribution distance metric (P-DDM) criterion. Then the robust effectiveness of domain distribution alignment is further improved by a fuzzy entropy regularization term. The proposed P-DDM is in theory proved which be an upper bound of the traditional distribution distance measure method MMD criterion under certain conditions. Therefore, minimizing P-DDM can effectively optimize the MMD objective. Secondly, based on the P-DDM criterion, a robust domain adaptation classifier based on P-DDM (C-PDDM) is proposed, which adopts the Laplacian matrix to preserve the geometric consistency of instances in the source domain and target domain for improving the label propagation performance. At the same time, by maximizing the use of source domain discriminative information to minimize domain discrimination error, the generalization performance of the learning model is further improved. Finally, a large number of experiments and analyses on multiple EEG datasets (i.e., SEED and SEED-IV) show that the proposed method has superior or comparable robustness performance (i.e., has increased by around 10%) in most cases.

## 1. Introduction

In the field of affective computing research (Mühl et al., 2014), automatic emotion recognition (AER) (Dolan, 2002) has received considerable attention from the computer vision community (Kim et al., 2013; Zhang et al., 2017). Thus far, numerous Electroencephalogram (EEG)-based emotion recognition methods have been proposed (Musha et al., 1997; Jenke et al., 2014; Zheng, 2017; Li X. et al., 2018; Pandey and Seeja, 2019). From a machine learning perspective, EEG-based AER can be modeled as a classification or regression problem (Kim et al., 2013; Zhang et al., 2017), where state-of-the-art AER techniques typically train their classifiers on multiple subjects to achieve accurate emotion recognition. In this case, subject-independent classifiers usually have poor generalization performance, as emotion patterns may vary across subjects (Pandey and Seeja, 2019). Significant progress in emotion recognition has been made by improving feature representation and learning models (Zheng et al., 2015; Zheng and Lu, 2015; Li et al., 2018a,b, 2019; Song et al., 2018; Du et al., 2020; Zhong et al., 2020). Since the individual differences in EEG-based AER are a natural existence, we may obtain a not good result by qualitative and empirical observations if the learned classifier generalize to previously unseen subjects (Jayaram et al., 2016; Zheng and Lu, 2016; Ghifary et al., 2017; Lan et al., 2019). As a possible solution, subject-specific classifiers are often impractical due to insufficient training data. Moreover, even if they are feasible in some specific scenarios, it is also an indispensable task to fine-tune the classifier to maintain a sound recognition capacity partly because the EEG signals of the same subject are changing now and then (Zhou et al., 2022). To address the aforementioned challenges, the domain adaptation (DA) learning paradigm (Patel et al., 2015; Tao et al., 2017, 2021, 2022; Zhang et al., 2019b; Dan et al., 2022) has been proposed and has achieved widespread effective applications, which enhances learning performance in the target domain by transferring and leveraging prior knowledge from other related but differently distributed domains (referred to as source or auxiliary domains), where the target domain has few or even no training samples.

Reducing or eliminating distribution differences between different domains is a crucial challenge currently faced during DA learning. To this end, mainstream DA learning methods primarily eliminate distribution biases between different domains by exploring domain-invariant features or samples (Pan and Yang, 2010; Patel et al., 2015). In order to fully exploit domain-invariant feature information, traditional shallow DA models have been extended to the deep DA paradigm. Benefiting from the advantages of deep feature transformation, deep DA methods have now achieved exciting adaptation learning performance (Long et al., 2015, 2016; Ding et al., 2018; Chen et al., 2019; Lee et al., 2019; Tang and Jia, 2019). Unfortunately, these deep DA methods can provide more transferable features and domain-invariant features, they can only alleviate but not eliminate the domain distribution shift problem caused by domain distribution differences. In addition, these deep DA methods can demonstrate better performance advantages, which may be attributed to one or several factors such as deep feature representation, model fine-tuning, adaptive regularization layers/terms, etc. However, the learning results of these methods still lack theoretical or practical interpretability at present.

DA theoretical studies have been proposed for domain adaptation generalization error bound (Ben-David et al., 2010) by the following inequality:

(1) $$\begin{array}{l} e_{T}\left(h\right)\leq e_{S}\left(h\right)+d_{H}\left(D_{S},D_{T}\right)+\\ \min\left\{\begin{array}{l} \varepsilon_{D_{S}}\left[\left|f_{S}\left(x\right)-f_{T}\left(x\right)\right|\right],\\ \varepsilon_{D_{T}}\left[\left|f_{S}\left(x\right)-f_{T}\left(x\right)\right|\right] \end{array}\right\} \end{array},$$

where the expected error of the target hypothesis $e_{T}\left(h\right)$ is mainly constrained by three aspects: (1) the expected error of the source domain hypothesis $e_{S}\left(h\right)$ ; (2) the distribution difference between the source and target domains $d_{H}\left(D_{S},D_{T}\right)$ ; (3) the difference in label functions between the two domains [i.e., the third term from Equation (1)]. Therefore, we will consider the three aspects simultaneously in this paper to reduce the domain adaptation generalization error bound (Zhang et al., 2021). Most existing methods assume that once the domain difference is minimized, a classifier trained only on the source domain can also generalize to the target domain well. Therefore, current mainstream DA methods aim to minimize the statistical distribution difference between the two domains. To this end, reducing or eliminating the distribution difference between domains to achieve knowledge transfer from the source domain and improve learning performance in the target domain is the core goal of domain adaptation learning methods. However, the key to this goal is effectively measuring the distribution difference between domains. Existing criteria for measuring the distance between different domains mainly include Maximum Mean Discrepancy (MMD) (Gretton et al., 2007), Bregman divergence, Jensen-Shannon divergence, etc. MMD is the most commonly used domain distribution difference measurement criterion in existing research, which can be divided into two categories alignment method: based on distribution alignment (including instance re-weighting and feature transformation) and classification model alignment with some representative works (Gretton et al., 2007; Pan et al., 2011; Tao et al., 2012, 2015, 2016, 2019; Baktashmotlagh et al., 2013; Chu et al., 2013; Long et al., 2013; Ganin et al., 2016; Liang et al., 2018; Luo et al., 2020; Kang et al., 2022).

To address the domain distribution shifting phenomenon, early instance re-weighting methods calculate the probability of each instance belonging to the source or target domain by likelihood ratio estimation (i.e., the membership of each instance). The domain shift problem can be relieved by re-weighting instances based on their membership. MMD (Gretton et al., 2007) is a widely adopted strategy for instance re-weighting, which is simple and effective. However, its optimization process is often carried out separately from the classifier training process, it’s difficult to ensure that both are optimal at the same time. To address this issue, Chu et al. (2013) proposed a joint instance re-weighting DA classifier. To overcome the conditional distribution consistency assumption of the instance re-weighting method, the feature transformation methods have received widespread attention and exploration (Pan et al., 2011; Baktashmotlagh et al., 2013; Long et al., 2013; Liang et al., 2018; Luo et al., 2020; Kang et al., 2022). Representative methods include Pan et al. (2011) proposed the Transfer Component Analysis (TCA) method, which learned a transformation matrix. It adopted MMD technology to minimize the distribution distance between source domains and target domain, and preserved data divergence information, but did not consider domain semantic realignment. Then, Long et al. (2013) proposed a Joint DA (JDA) method, which fully considered the domain feature distribution alignment and class conditional distribution alignment with the target domain labels in the class conditional distribution initialized by pseudo-labels. Recently, Luo et al. (2020) proposed a Discriminative and Geometry Aware Unsupervised Domain Adaptation (DGA-DA) framework, which combined the TCA and JDA methods. It introduced a strategy that made different classes from cross-domains mutually exclusive. Most of the existing affective models were based on deep transfer learning methods built with domain-adversarial neural network (DANN) (Ganin et al., 2016) proposed in Li et al. (2018c,d), Du et al. (2020), Luo et al. (2018), and Sun et al. (2022). The main idea of DANN (Ganin et al., 2016) was to find a shared feature representation for the source domain and the target domain with indistinguishable distribution differences. It also maintained the predictive ability of the estimated features on the source samples for a specific classification task. In addition, the framework preserved the geometric structure information of domain data to achieve effective propagation of target labels. Baktashmotlagh et al. (2013) proposed a Domain Invariant Projection (DIP) algorithm, which investigated the use of polynomial kernels in MMD to construct a compact domain-shared feature space. The series of DANN methods still has some challenges, PR-PL (Zhou et al., 2022) also explored the prototypical representations to further characterize the different emotion categories based on the DANN method. Finally, the study designed a clustering-based DA concept to minimize inner-class divergence. A review of existing DA method research shows that MMD is the main distribution distance measurement technique adopted by feature transformation-based DA methods. Traditional MMD-based DA methods focused solely on minimizing cross-domain distribution differences while ignoring the statistical (clustering) structure of the target domain distribution, which to some extent affects the inference of target domain labels. To address this issue, Kang et al. (2022) proposed a contrastive adaptation network based on unsupervised domain adaptation. The initialization of the labels from the target domain was realized by the clustering assumption. The feature representation is adjusted by measuring the contrastive domain differences (i.e., minimizing within-class domain differences and maximizing between-class domain differences) in multiple fully connected layers. During the training process, the assumptions of the target domain label and the feature representations are continuously cross-iterated and optimized to enhance the model’s generalization capability. Furthermore, inspired by clustering patterns, Liang et al. (2018) proposed an effective domain-invariant projection integration method that uses clustering ideas to seek the best projection for each class within the domain, bridging the domain-invariant semantic gap and enhance the inner-class compactness in the domain. However, it still essentially belongs to MMD-based feature transformation DA methods.

**Figure 1:**
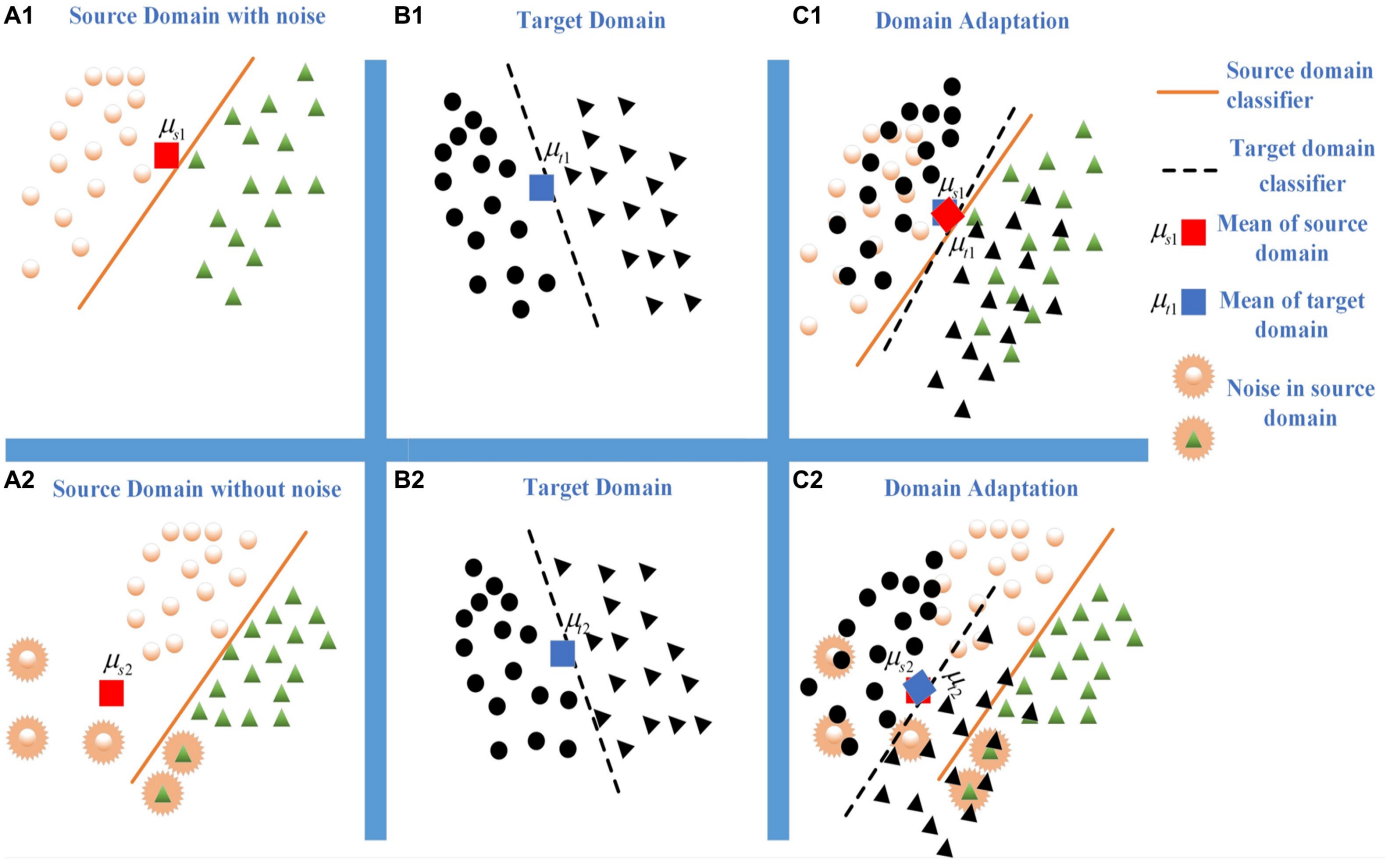
The influence comes from the noises or outliers during domain matching. **(A1)** Source domain with noise. **(B2)** Target domain. **(C3)** Domain adaptation. **(A1)** Source domain without noise. **(B2)** Target domain. **(C3)** Domain adaptation.

It is worth noting that existing MMD-based methods did not fully consider the impact of intra-domain noise when measuring domain distribution distance. In real scenarios, noise inherently exists in domains, and intra-domain noise can lead to mean-shift problems in distance measurement for traditional MMD methods and their variants. This phenomenon to some extent is affecting the generalization performance of MMD-based DA methods. As shown in Figures 1A1, B1 represent the noise-free source domain and target domain, respectively. $\mu_{s*}$ and $\mu_{t*}$ are the means of the source domain and target domain, respectively. Figure 1C1 shows the domain adaptation result based on the MMD method. When the source domain has noises (i.e., Figure 1A2), the mean shift occurs and it’s difficult to effectively measure the distribution distance by the MMD criterion. It matches the most of target domain samples (i.e., Figure 1B2) to a certain category of source domain (i.e., Figure 1C2). It declines the inferring performance of domain adaptation learning.

Existing research (Krishnapuram and Keller, 1993) pointed out that the possibilistic-based clustering model can effectively suppress noise interference during the clustering process. Therefore, Dan et al. (2021) proposed an effective classification model based on the possibilistic clustering assumption. Inspired by this work, we aim to jointly address the robustness and discriminative issues in the MMD criterion to enhance the adaptability of MMD-based methods and propose a robust Probabilistic Distribution Distance Measure (P-DDM) criterion. Specifically, by measuring the distance between EEG data (from either the source or target domain) and the overall domain mean (i.e., the mean of the source domain and target domain), the corresponding matching membership is used to judge the relevance between the EEG data and the mean. In other words, the smaller the distance between the EEG data and the mean, the larger the membership, and vice versa. In this way, the impact of noise in the matching process can be alleviated by the value of membership. The robustness and effectiveness of P-DDM are further enhanced by introducing a fuzzy entropy regularization term. Based on this, a domain adaptation Classifier model based on P-DDM (C-PDDM) is proposed, which introduces the graph Laplacian matrix to preserve the geometric structure consistency within the source domain and target domain. It can improve the label propagation performance. At the same time, a target domain classification model with better generalization performance is obtained by maximizing the use of source domain discriminative information to minimize domain discriminative errors. The main contributions of this paper are as follows:

1. The traditional MMD measurement is transformed into a clustering optimization problem, and a robust possibilistic distribution distance metric criterion (P-DDM) is proposed to solve the domain mean-shift problem in a noisy environment;
2. It is theoretically proven that under certain conditions, P-DDM is an upper bound of the traditional MMD measurement. The minimization of MMD in domain distribution measurement can be effectively achieved by optimizing the P-DDM;
3. A DA classifier mode based on P-DDM is proposed (i.e., C-PDDM), its consistent convergence is proven, and the DA generalization error bound of the method is proposed based on Rademacher complexity theory;
4. A large number of experiments are conducted on two EEG datasets (i.e., SEED and SEED-IV), demonstrating the robust effectiveness of the method and a certain degree of improvement in the classification accuracy of the model.

## 2. Proposed framework: C-PDDM

In domain adaptation learning, $D_{S}={\left\{x_{i}^{s},y_{i}^{s}\right\}}_{i=1}^{n}$ denotes n samples and its associated labels of the source domain. $X^{s}=\left[x_{1}^{s},\ldots ,x_{n}^{s}\right]\in {\mathbb{R}}^{d\times n}$ indicates all the source samples. $Y^{s}={\left\{y_{1},\ldots {\mathrm{,y}}_{n}\right\}}^{T}\in {\left\{0,1\right\}}^{n\times C}$ is the associated labels with a one-hot coding vector $y_{i}\in {\mathbb{R}}^{C}\left(1\leq i\leq n\right)$ . If $x_{i}$ belongs to the j-*th* class, The other elements $y_{i}$ are zero. $D_{T}={\left\{x_{j}^{t}\right\}}_{j=1}^{m}$ denotes the target domain with no label, which $X^{t}=\left[x_{1}^{t},\ldots {\mathrm{,x}}_{m}^{t}\right]\in {\mathbb{R}}^{d\times m}$ means *m* data points. $Y^{t}={\left\{y_{1},\ldots ,y_{m}\right\}}^{T}\in {\mathbb{R}}^{m\times C}$ is unknown during training. Let $X=\left[X^{s},X^{t}\right]\in {\mathbb{R}}^{d\times N}$ , N=n+m , $\mu_{s}=\frac{1}{n}\overset{n}{\underset{i=1}{\sum }}x_{i}^{s}$ , and $\mu_{t}=\frac{1}{m}\overset{m}{\underset{j=1}{\sum }}x_{j}^{t}$ denotes the mean value of the source domain and target domain, respectively. Our proposal has some assumptions:

1. However, the distributions of source domain ( ℙ ) and target domain ( ℚ ) are different (i.e., $\mathbb{P}\left(X_{S}\right)\neq \mathbb{Q}\left(X_{T}\right)$ and $X_{S}=X_{T}$ ), they share the same feature space with $X_{S},X_{T}\in X$ are feature space of the source domain and target domain, respectively.
2. The condition probability distributions of the source domain and target domain are different [i.e., $\mathbb{P}\left(Y_{S}|X_{S}\right)\neq \mathbb{Q}\left(Y_{T}|X_{T}\right)$ ], but they share the same label space with $Y_{S},Y_{T}\in Y$ are label space of the source domain and target domain, respectively.

In the face of a complex and noisy DA environment, the proposed method will achieve the following objectives by the DA generalization error theory (Ben-David et al., 2010) to make the distance metric for domain adaptation more robust and achieve good target classification performance: (1) Robust distance metric: solve the problem of domain mean shift under the influence of noise, thereby effectively aligning the domain distribution differences; (2) Implement target domain knowledge inference: we bridge the discriminative information of the source domain while minimizing the domain discriminative error based on preserving the consistency of domain data geometry, and learn a target domain classification machine with high generalization performance. Based on the descriptions of the above objectives, the general form of the proposed method can be described as:


(2) $$\Theta \left(\lambda_{i},Y,W\right)=\min\Omega \left(\lambda_{k},X^{s},X^{t}\right)+R\left(Y,W\right)$$

where $\Omega \left(\lambda_{k},X^{s},X^{t}\right)$ is the robust distance metric, which reduces the impact of noisy data on the alignment of domain distribution differences. *R(Y, W)* is the domain adaptation learning loss function that includes the label matrix *Y* (that is, the comprehensive label matrix of the source and target domains) and the comprehensive learning model *W* of the source domain and the target domain.

### 2.1. Design of possibilistic distribution distance metric

#### 2.1.1. Motivation

In a certain reproducing kernel Hilbert space (RKHS) H , the original space data representation can be transformed into a feature representation in the RKHS through a certain non-linear transformation $\phi :{\mathbb{R}}^{d}\to H$ (Long et al., 2016). The corresponding kernel function is defined as $K\left(X_{1},X_{2}\right):X\times X\to \mathbb{R}$ , where $K\left(x_{1},x_{2}\right)={\langle \phi \left(x_{1}\right),\phi \left(x_{2}\right)\rangle }_{H}$ , $x_{1},x_{2}\in X$ . It is also a commonly used kernel technique in current non-linear learning methods (Pan et al., 2011; Long et al., 2015). For the problem of inconsistent distributions in domain adaptation, existing research has shown (Bruzzone and Marconcini, 2010; Gretton et al., 2010) that when sample data is mapped to a high-dimensional or even infinite-dimensional space, it can capture higher-dimensional feature representations of the data (Carlucci et al., 2017). That is, in a certain RKHS, the distance between two distributions can be effectively measured through the maximum mean discrepancy (MMD) criterion. Based on this, it is assumed that F is a collection of functions of a certain type f : f:X→ℝ , The maximum mean discrepancy (MMD) between two domain distributions ℙ and ℚ can be defined as:

(3) $$MMD_{F}\left[\mathbb{P},\mathbb{Q}\right]:=\underset{f\in F}{\sup}\left(\underset{\mathbb{P}}{E}\left[f\left(x\right)\right]-\underset{\mathbb{Q}}{E}\left[f\left(x\right)\right]\right).$$

MMD measure minimizes the expected difference between two domain distributions through the function *f*, making the two domain distributions as similar as possible. When the sample size of the domain is sufficiently large (or approaches infinity), the expected difference approximates (or equals) the empirical mean difference. Therefore, Equation (3) can be written in the empirical form of MMD:

(4) $$MMD\left(X^{s},X^{t}\right):={\left\|\frac{1}{n}\overset{n}{\underset{i=1}{\sum }}\phi \left(x_{i}^{s}\right)-\frac{1}{m}\overset{m}{\underset{j=1}{\sum }}\phi \left(x_{j}^{t}\right)\right\|}_{H}^{2}.$$

To prove the universal connection between the traditional MMD criterion and the mean clustering model, we give the following theorem:

**Theorem 1**. The MMD measure can be loosely modeled as a special clustering problem with one cluster center, where the clustering center is μ , and the instance clustering membership is $\varsigma_{k}$ .

**Proof:** As defined by MMD:

(5) $$\begin{array}{l} MMD\left(D^{s},D^{t}\right)\\ ={\left\|\frac{1}{n}\sum_{i=1}^{n}\phi \left(x_{i}^{s}\right)-\frac{1}{m}\sum_{j=1}^{m}\phi \left(x_{j}^{t}\right)\right\|}_{H}^{2}\\ ={\left\|\frac{1}{n}\sum_{i=1}^{n}\phi \left(x_{i}^{s}\right)-\mu +\mu -\frac{1}{m}\sum_{j=1}^{m}\phi \left(x_{j}^{t}\right)\right\|}_{H}^{2}\\ \leq \left\|\frac{1}{n}\sum_{i=1}^{n}\phi \left(x_{i}^{s}\right)-\mu \right\|+{\left\|\frac{1}{m}\sum_{j=1}^{m}\phi \left(x_{j}^{t}\right)-\mu \right\|}_{H}^{2}\\ =\frac{1}{n^{2}}\left\|\sum_{i=1}^{n}\phi \left(x_{i}^{s}\right)-n\mu \right\|+\frac{1}{m^{2}}{\left\|\sum_{j=1}^{m}\phi \left(x_{j}^{t}\right)-m\mu \right\|}_{H}^{2}\\ =\frac{1}{n^{2}}{\left\|\sum_{i=1}^{n}\left(\phi \left(x_{i}^{s}\right)-\mu \right)\right\|}_{H}^{2}+\frac{1}{m^{2}}{\left\|\sum_{j=1}^{m}\left(\phi \left(x_{j}^{t}\right)-\mu \right)\right\|}_{H}^{2}\\ \leq \frac{1}{n^{2}}{\sum_{i=1}^{n}\left\|\phi \left(x_{i}^{s}\right)-\mu \right\|}_{H}^{2}+\frac{1}{m^{2}}\sum_{j=1}^{m}{\left\|\phi \left(x_{j}^{t}\right)-\mu \right\|}_{H}^{2}\\ =\sum_{k=1}^{N}\varsigma_{k}{\left\|\phi \left(x_{k}\right)-\mu \right\|}_{H}^{2} \end{array}$$

where $\mu =\delta \mu_{s}+\left(1-\delta \right)\mu_{t}$ is the cluster center with 0≤δ≤1 . When n=m , let. When n≠m , the number of samples in the source domain and target domain can be set the same during sampling. The sample membership $\varsigma_{k}$ of one cluster center is defined as:

(6) $$\varsigma_{k}=\{\begin{array}{l} \frac{1}{n^{2}},\;\;x_{k}\in X^{s}\\ \frac{1}{m^{2}},\;\;x_{k}\in X^{t} \end{array}.$$

From Equation (5), it can be seen that the one cluster center form with clustering center n is an upper bound of the traditional MMD measure. In other words, the MMD measure can be relaxed to a special one cluster center objective function. By optimizing this clustering objective, the minimization of MMD between domains can be achieved.

As indicated in Theorem 1 and Baktashmotlagh et al. (2013), the domain distribution MMD criterion is essentially related to the clustering model, which can be used to achieve more effective distribution alignment between different domains by clustering domain data. It is worth noting that the traditional clustering model has the disadvantage of being sensitive to noise (Krishnapuram and Keller, 1993), which makes domain adaptation (DA) methods based on MMD generally face the problem of domain mean shift caused by noisy data. To address this issue, this paper further explores more robust forms of clustering and proposes an effective new criterion for domain distribution distance measurement.

#### 2.1.2. P-DDM

Recently proposed possibility clustering models can effectively overcome the impact of noise on clustering performance (Dan et al., 2021). Therefore, this paper further generalizes the above special one cluster center to a possibility one cluster center form and proposes a robust possibility distribution distance metric criterion P-DDM. By introducing the possibility clustering assumption, the MMD hard clustering form is generalized to a soft clustering form, which controls the contribution of each instance according to its distance from the overall domain mean. The farther the distance, the smaller the contribution of the instance, thus weakening the influence of mean shift caused by noisy data in the domain and improving the robustness of domain adaptation learning.

To achieve robust domain distribution alignment, the distribution distance measurement criterion based on the possibility clustering assumption mainly achieves two goals: (1) Calculate the difference in distribution between kernel space domains based on the possibility clustering assumption, by measuring the distance between each instance in the domain and the overall domain mean; (2) Measure the matching contribution of each instance. Any instance in the overall domain has a matching contribution value $\lambda_{k}\in \mathbb{R}$ , k=1,2,…N , which is the matching contribution degree of $x_{k}$ to the overall domain mean, and the closer the distance, the larger the value of $\lambda_{k}$ . Thus, the possibility distribution distance measure can be defined as:

(7) $$\begin{array}{l} \Omega_{p}\left(\lambda_{k},X_{s},X_{t}\right)=\overset{N}{\underset{k=1}{\sum }}\lambda_{k}^{b}{\left\|\phi \left(x_{k}\right)-\mu \right\|}_{H}^{2}\\ s.t.,0\leq \lambda_{k}\leq 1,k=1,\ldots ,N \end{array},$$

where the parameter *b* is the weight exponent of $\lambda_{k}$ , which is used to adjust the uncertainty or degree of the data points belonging to multiple categories. In order to circumvent the trivial solution, *b* is set to 2 in the subsequent equations of this paper. The detailed process of different values of *b* can be found in references (Krishnapuram and Keller, 1993). $\Omega_{p}\left(\lambda_{k},X_{s},X_{t}\right)$ is an objective function of possibility clustering with a cluster center of *μ*, and when $\lambda_{k}^{2}=\varsigma_{k}$ , $\Omega_{p}\left(\lambda_{k},X_{s},X_{t}\right)$ takes the form of the above-mentioned special one cluster center.

Theorem 2. When $\lambda_{k}\in \left[\frac{1}{r},1\right]$ , the possibility distribution distance measure $\Omega_{p}\left(\lambda_{k},X_{s},X_{t}\right)$ is an upper bound of the traditional MMD method.

**Proof:** Combining Equation (5) and Equation (7), we have the following inference process:

(8) $$\begin{array}{l} \underset{K}{\min}MMD\left(X^{s},X^{t}\right)\\ \leq \overset{N}{\underset{k=1}{\sum }}\varsigma_{k}{\left\|\phi \left(x_{k}\right)-\mu \right\|}_{H}^{2}\\ \leq \overset{N}{\underset{k=1}{\sum }}\lambda_{k}^{2}{\left\|\phi \left(x_{k}\right)-\mu \right\|}_{H}^{2}\\ =\Omega_{p}\left(\lambda_{k},X_{s},X_{t}\right) \end{array}$$

According to the value range of $\varsigma_{k}$ , when λk∈1r,1 and *r* = *min* (*n*, *m*), the second inequality in Equation (8) holds, thus proving that $\Omega_{p}\left(\lambda_{k},X_{s},X_{t}\right)$ is the upper bound of traditional MMD. According to Theorem 1 and Theorem 2, the traditional MMD metric criterion can be modeled as a possibilistic one cluster center objective form. From this perspective, it can be considered that the possibilistic distribution distance metric target domain can not only achieve alignment of domain feature distribution, but also weaken the “negative transfer” effect of noisy data in the domains during training.

Equation (7) only considers the overall mean regression problem, which clusters each instance with the overall domain mean, while ignoring the semantic structural information of the instance in domain distribution alignment. It may lead to the destruction of the local class structure in the domain. Inspired by the idea of global and local from Tao et al. (2016), we further consider the semantic distribution structure in domain alignment and calculate the semantic matching contribution of each instance. Therefore, based on the feature distribution alignment, we propose an integrated semantic alignment. It can be rewritten as follows:

(9) $$\begin{array}{l} \Omega_{pc}\left(\lambda_{k},X_{s},X_{t}\right)\\ =\underset{\lambda_{k,c}}{\min}\overset{C}{\underset{c=0}{\sum }}\overset{N}{\underset{k=1}{\sum }}\lambda_{k,c}^{2}{\left\|\phi \left(x_{k,c}\right)-\mu_{c}\right\|}_{H}^{2}\\ s.t.,0\leq \lambda_{k,c}\leq 1 \end{array}$$

where $\mu_{c}=\delta \mu_{s,c}+\left(1-\delta \right)\mu_{t,c}$ , $\mu_{s,c}=\frac{1}{n}\overset{C}{\underset{c=0}{\sum }}\overset{n_{c}}{\underset{i=1}{\sum }}\phi \left(x_{i,c}^{s}\right)$ , $\mu_{t,c}=\frac{1}{m}\overset{C}{\underset{c=0}{\sum }}\overset{m_{c}}{\underset{j=1}{\sum }}\phi \left(x_{j,c}^{t}\right)$ , c=0,1,2,…,C , C is the number of classes. $n_{c}$ is the number of samples of the *c-th* class in the source domain, $m_{c}$ is the sample number of the *c-th* class in the target domain, and $n=\overset{C}{\underset{c=0}{\sum }}n_{c}$ , $m=\overset{C}{\underset{c=0}{\sum }}m_{c}$ . When $m=\overset{C}{\underset{c=0}{\sum }}m_{c}$ . When c=0 , $\mu_{s,c}$ and $\mu_{t,c}$ are the mean values of the source domain and the target domain, respectively. Equation (9) is a feature distribution alignment form. When c∈[1,2,…,C] , $\mu_{s,c}$ and $\mu_{t,c}$ are the associated *c-th* class mean values of the source domain and the target domain, respectively. $\lambda_{k,c}$ is the membership of $x_{k}$ belonging to the *c-th* class in the overall domain (i.e., integrate the source domain and target domain into one domain).

To further improve the robustness and effectiveness of the possibilistic distribution distance metric method on noisy data, we add a fuzzy entropy regularization term related in Equation (9). Therefore, the semantic alignment P-DDM in (9) can be further defined as follows:

(10) $$\begin{array}{l} \begin{array}{l} \Omega \left(\lambda_{k},X_{s},X_{t}\right)\\ =\overset{C}{\underset{c=0}{\sum }}\overset{N}{\underset{k=1}{\sum }}\lambda_{k,c}^{2}{\left\|\phi \left(x_{k,c}\right)-\mu_{c}\right\|}_{H}^{2}\\ +\beta \overset{C}{\underset{c=0}{\sum }}\overset{N}{\underset{k=1}{\sum }}\left(\lambda_{k,c}^{2}\ln\lambda_{k,c}^{2}-\lambda_{k,c}^{2}\right) \end{array},\\ s.t.,0\leq \lambda_{k,c}\leq 1 \end{array}$$

where β is a tunable balancing parameter that forces the value of $\lambda_{k,c}$ for relevant data to be as large as possible to avoid trivial solutions. After the above improvements, P-DDM is a monotonic decreasing function on $\lambda_{k,c}$ . Through the fuzzy entropy term in the second part of Equation (10), P-DDM reduces the impact of noise data on model classification. The larger the fuzzy entropy, the greater the sample discrimination information, which helps to enhance the robustness and effectiveness of distribution distance measurement. Additionally, the possibility distribution measurement model regularized by fuzzy entropy can effectively suppress the contribution of noise data in domain distribution alignment, thereby reducing the interference of noise/abnormal data to domain adaptation learning. The robustness effect of fuzzy entropy can be further seen in the empirical analysis of reference (Gretton et al., 2010).

### 2.2. Design of domain adaptation function

The P-DDM criterion addresses the problems of domain distribution alignment and noise impact. Next, we will achieve the two goals required for the inference of target domain knowledge: (1) to preserve the geometric consistency in the source domain and the target domain, i.e., the label information between adjacent samples should be consistent, and (2) to minimize the structural risk loss of both the source and target domains. Given the description of the objective task, the general form of the objective risk function can be described as:

(11) $$R\left(Y,W\right)=\Omega_{Y}+\Omega_{W},$$

where $\Omega_{Y}$ is the loss of joint knowledge transfer and label propagation, which preserves the geometric consistency of the data between the source and target domains, and $\Omega_{W}$ is the structural risk loss term, which includes both the source domain and the target domain. Next, these two terms will be designed separately.

#### 2.2.1. Joint knowledge transfer and label propagation

Firstly, G=〈X,M〉 denotes an undirected weighted graph of the overall domain. $M\in {\mathbb{R}}^{N\times N}$ is a weighted matrix with $M_{ij}=M_{ji}\geq 0$ . $M_{ij}$ is calculated by:

(12) $$M_{ij}=\{\begin{array}{c} \exp\left(-\frac{{\left\|x_{i}-x_{j}\right\|}^{2}}{2\sigma^{2}}\right)\\ 0,\;\mathrm{otherwise}\; \end{array},x_{i}\in Ne\left(x_{j}\right)或x_{j}\in Ne\left(x_{i}\right),$$

where $x_{k}\in Ne\left(x_{m}\right)$ means that $x_{k}$ is the neighbor of $x_{m}$ . σ is the local influence range parameter that controls the Gaussian kernel function and is also a hyper-parameter. The larger the value of σ , the larger the local influence range, and vice versa, the smaller the local influence range. When σ is fixed, the change in $M_{ij}$ decreases monotonically as the distance between $x_{i}$ and $x_{j}$ increases.

In combination with source domain knowledge transfer and graph Laplacian matrix (Long et al., 2013; Wang et al., 2017), the objective form of label propagation modeling can be described as:

(13) $$\Omega_{Y}=\underset{Y}{\min}\mathrm{tr}\left(Y^{T}\mathrm{LY}\right),$$

where $Y=\left[Y_{s}{\mathrm{;Y}}_{t}\right]\in {\mathbb{R}}^{N\times C}$ , $Y_{t}$ is the target domain label matrix. The label value for a sample in the target domain corresponding to a position in $Y_{t}$ is all zeros when the sample has no label. $Y_{s}$ is the source domain label matrix. $L=M-D\in {\mathbb{R}}^{N\times N}$ is the Laplacian graph matrix (Long et al., 2013) with D is a diagonal matrix and $D_{ii}=\overset{N}{\underset{j=1}{\sum }}M_{ij}$ .

#### 2.2.2. Minimize structural risk loss

In our proposed method, the classifier of the source domain (the corresponding target domain classification model) is defined as $f_{s}=W_{ss}{}^{T}X_{s}+b_{s}$ (the corresponding $f_{t}=W_{tt}{}^{T}X_{t}+b_{t}$ ). $b_{s}$ ( $b_{t}$ ) is the source domain bias (the target source bias). $W_{ss}$ ( $W_{tt}$ ) is the parameter matrix of the source domain (the parameter matrix of the target domain). Let $\tilde{W_{s}}=\left[W_{ss},b_{s}\right]$ , $\tilde{X_{s}}=\left[X_{s},1\right]$ , $\tilde{W_{t}}=\left[W_{tt},b_{t}\right]$ , $\tilde{X_{t}}=\left[X_{t},1\right]$ , we can rewrite both classifiers of the source domain and the target domain respectively: $\tilde{f_{s}}={\tilde{W_{s}}}^{T}\tilde{X_{s}}$ and $\tilde{f_{t}}={\tilde{W_{t}}}^{T}\tilde{X_{t}}$ . Let $W=\left[\tilde{W_{s}},\tilde{W_{t}}\right]$ , $X=\left[\tilde{X_{s}},\tilde{X_{t}}\right]$ . We rewrite the final classifier as: $F\left(W\right)=X^{T}W$ .

According to the minimum square loss function, the problem of minimizing structural risk loss in both domains (source domain and target domain) can be described as:

(14) $$\Omega_{W}=\overset{C}{\underset{c=0}{\sum }}\overset{N}{\underset{k=1}{\sum }}\lambda_{k,c}^{2}{\left\|\phi^{T}\left(x_{k}\right)W-y_{k}\right\|}_{H}^{2}+\rho {\left\|W\right\|}_{2,1}$$

where the first term denotes the structure risk loss and $y_{k}\in Y$ The second term is the constraint term of *W*. By using $l_{2,1}$ regularization, we can achieve feature selection and it can effectively control the complexity of the model to prevent over-fitting of the target classification model to some extent.

The classification task proposed in this method is ensured by the dual prediction of the label matrix *Y* and the decision function *W* to guarantee the reliability of the prediction. The target classification function is combined by Equation (13) and Equation (14). It’s described as follows:

(15) $$\begin{array}{l} R\left(Y,W\right)=\alpha tr\left(Y^{T}LY\right)+\\ \overset{C}{\underset{c=0}{\sum }}\overset{N}{\underset{k=1}{\sum }}\lambda_{k,c}^{2}{\left\|\phi^{T}\left(x_{k}\right)W-y_{k}\right\|}_{H}^{2}+\rho {\left\|W\right\|}_{2,1}\\ s.t.,0\leq \lambda_{k,c}\leq 1,YY^{T}=I \end{array},$$

### 2.3. Final formulation

By combining the semantic alignment P-DDM form [i.e., Equation (10)] and the target classification function [i.e., Equation (16)], the final optimization problem formulation of the proposed method C-PDDM can be described as follows:

(16) $$\begin{array}{c} \Theta \left(\lambda_{k},Y,W\right)\\ =\begin{array}{c} \min\\ \lambda_{k},Y,W \end{array}\overset{C}{\underset{c=0}{\sum }}\overset{N}{\underset{k=1}{\sum }}\lambda_{k,c}^{2}{\left\|\phi \left(x_{k,c}\right)-\mu_{c}\right\|}_{H}^{2}\\ \begin{array}{c} +\beta \overset{C}{\underset{c=0}{\sum }}\overset{N}{\underset{k=1}{\sum }}\left(\lambda_{k,c}^{2}\ln\lambda_{k,c}^{2}-\lambda_{k,c}^{2}\right)+\alpha tr\left(Y^{T}LY\right),\\ +\overset{C}{\underset{c=0}{\sum }}\overset{N}{\underset{k=1}{\sum }}\lambda_{k,c}^{2}{\left\|\phi^{T}\left(x_{k}\right)W-y_{k}\right\|}_{H}^{2}+\rho {\left\|W\right\|}_{2,1},\\ s.t.,0\leq \lambda_{k,c}\leq 1,YY^{T}=I \end{array} \end{array}$$

where β , α , and ρ are balance parameters.

With all model parameters obtained, target domain knowledge inference can be achieved by maximizing the utilization of source domain discriminative information, linearly fusing the two classifiers $\tilde{f_{s}}$ and $\tilde{f_{t}}$ , and using this linear fusion model for target domain knowledge inference. The fusion form can be written as follows:

$$j=\arg\underset{j}{\max}{\left(y_{i}^{t}=\upsilon \tilde{f_{s}}(x_{i}^{t})+(1-\upsilon )\tilde{f_{t}}(x_{i}^{t})\right)}_{j}$$

where υ∈[0,1] is an adjustable parameter that balances the two classifiers, in order to reflect the importance of source domain discriminative information as prior knowledge, υ is set to 0.9 based on empirical experience.

## 3. C-PDDM optimization

The optimization problem of C-PDDM is a non-convex problem with respect to $\lambda_{k,c}$ , W, and Y. We will adopt an alternating iterative optimization strategy to achieve the optimization and solution of $\lambda_{k,c}$ , W, and Y, so that each optimization variable has a closed-form solution.

### 3.1. Update ***λ***_k,c_ as given ***W*** and ***Y***

As we fix W and Y, the objective function in Equation (16) reduces to solving:

(17) $$\begin{array}{l} \underset{}{\underset{\lambda_{k,c}}{\min}P_{1}=\min}\sum_{c=0}^{C}\sum_{k=1}^{N}\lambda_{k,c}^{2}{\left\|\phi \left(x_{i,c}^{}\right)-\mu_{c}^{}\right\|}_{H}^{2}\\ -\beta \sum_{c=0}^{C}\sum_{k=1}^{N}(-\lambda_{k,c}^{2}\ln\lambda_{k,c}^{2}+\lambda_{k,c}^{2})+\\ +\sum_{c=0}^{C}\sum_{k=1}^{N}\lambda_{k,c}^{2}{\left\|\phi^{T}(x_{i})W-y_{i}\right\|}_{H}^{2}\\ s.t.0\leq \lambda_{k,c}^{}\leq 1 \end{array}$$

**Theorem 3**. The optimal solution to the primal optimization problem of the objective function (17) is:

(18) $$\lambda_{k,c}=\exp\left(-\frac{J}{\beta }\right),$$

where $J=\overset{C}{\underset{c=0}{\sum }}\overset{N}{\underset{k=1}{\sum }}{\left\|\phi^{T}\left(x_{k}\right)W-y_{k}\right\|}_{H}^{2}+\overset{C}{\underset{c=0}{\sum }}\overset{N}{\underset{k=1}{\sum }}{\left\|\phi \left(x_{k,c}\right)-\mu_{c}\right\|}_{H}^{2}.$

**Proof.** By setting the derivative ∂P1∂λk,c=0 , we obtain:

(19) $$\begin{array}{c} \frac{\partial P_{1}}{\partial \lambda_{k,c}}=2\overset{C}{\underset{c=0}{\sum }}\overset{N}{\underset{k=1}{\sum }}\lambda_{k,c}{\left\|\phi \left(x_{k,c}\right)-\mu_{c}\right\|}_{H}^{2}\\ \begin{array}{c} +2\beta \overset{C}{\underset{c=0}{\sum }}\overset{N}{\underset{k=1}{\sum }}\lambda_{k,c}\ln\lambda_{k,c}^{2}\\ +2\overset{C}{\underset{c=0}{\sum }}\overset{N}{\underset{k=1}{\sum }}\lambda_{k,c}{\left\|\phi^{T}\left(x_{k}\right)W-Y\right\|}_{H}^{2}=0 \end{array} \end{array}$$

Combining and simplifying the terms in Equation (19), we get the solution of $\lambda_{k,c}$ is Equation (18), Theorem 3 is proved. From Theorem 3, the membership of any sample can be obtained by Equation (18).

### 3.2. Update ***W*** as given ***Y*** and ***λ***_k,c_

Since the first and the third terms in Equation (16) do not have W, the optimization formula for C-PDDM can be rewritten as:

(20) $$\begin{array}{l} P_{2}=\underset{W}{\min}\overset{C}{\underset{c=0}{\sum }}\overset{N}{\underset{k=1}{\sum }}\lambda_{k,c}^{2}{\left\|\phi^{T}\left(x_{k}\right)W-y_{k}\right\|}_{H}^{2}+\rho {\left\|W\right\|}_{2,1}\\ =\underset{W}{\min}\lambda {\left\|\phi^{T}\left(X\right)W-Y\right\|}_{H}^{2}+\rho {\left\|W\right\|}_{2,1} \end{array},$$

where λ is a matrix with $\lambda \in {\mathbb{R}}^{N\times C}$ , each element is $\lambda_{k,c}^{2}$ , $\lambda_{k,c}$ means the membership of $x_{k}$ belonging to the *c-th* class.

Theorem 4. The optimal solution to the primal optimization problem of the objective function (20) is:

(21) W=AY,

with $A={\left(\lambda \phi \left(X\right)\phi^{T}\left(X\right)+\rho U\right)}^{-1}\phi \left(X\right)$ .

**Proof.** According to Equation (19), let ∂P2∂W=0 , we have:

(22) ∂P2∂W=2λ[ϕ(X)(ϕT(X)W−Y)]+2ρUW=0,

where $\frac{\partial \rho {\left\|W\right\|}_{2,1}}{\partial W}=UW$ , U is a diagonal matrix, its diagonal element is $U_{ii}=\frac{1}{\left\|w_{i}\right\|}$ , $w_{i}$ is the *i*-th vector of W . The solution obtained by organizing Equation (22) is Equation (21).

### 3.3. Update ***Y*** by fixing ***W*** and ***λ***_k,c_

Finally, $\lambda_{k,c}$ is fixed. W=AY is substituted into Equation (16). The constraint ${\mathrm{YY}}^{T}=I$ can reduce the interference information in the label matrix Y , the objective form for optimizing the solution of Y is described as:

(23) $$\begin{array}{l} P_{3}=\underset{Y^{T}Y=I}{\min}\alpha tr(Y^{T}LY)\\ {}_{}\overset{}{}+\sum_{c=0}^{C}\sum_{k=1}^{N}\lambda_{k,c}^{2}{\left\|\phi^{T}(x_{k})W-y_{k}\right\|}_{H}^{2}\\ {}_{}=\underset{Y^{T}Y=I}{\min}\alpha tr(Y^{T}LY)\\ {}_{}\overset{}{}+\lambda {\left\|\phi^{T}(X)AY-Y\right\|}_{H}^{2}\\ {}_{}=\underset{Y^{T}Y=I}{\min}tr\left(Y^{T}HY\right) \end{array}$$

where $H=\alpha L+\lambda B^{T}B$ , $B=\phi^{T}\left(X\right)A-I$ .

The optimization problem (23) is a standard singular value decomposition problem, where Y is the eigenvector of the matrix H . Y can be obtained by solving the singular value decomposition of the matrix H .

## 4. Algorithm

### 4.1. Algorithm description

In unsupervised domain adaptation learning scenarios (i.e., the target domain does not have any labeled data), in order to achieve semantic alignment between domains, initial labels of the target domain can be obtained through three strategies (Liang et al., 2018): (1) random initialization; (2) zero initialization; (3) use the model trained on the source domain data to cluster the target domain data to obtain initial labels. (1) and (2) belong to the cold-start method. (3) belongs to the hot-start method which is relatively friendly to subsequent learning performance. Therefore, we adopt the third method to initialize the prior information of $\lambda_{k,c}$ , W , and Y . The proposed method adopts the iterative optimization strategy commonly used in multi-objective optimization, and the algorithm stops iterating when the following conditions are satisfied: $\left|\Theta \left(\lambda_{k,c}^{z},W^{z},Y^{z}\right)-\Theta (\lambda_{k,c}^{z-1},,W^{z-1},,Y^{z-1})\right|<\varepsilon ,$ where $\Theta \left(\lambda_{k,c}^{z},W^{z},Y^{z}\right)$ denotes the value of the objective function at the *z-th* iteration. ε is a pre-defined threshold.

**Table:** 

ALGORITHM 1 Domain adaptation learning based on C-PDDM.**Input:** The source domain data $\left\{X_{s},Y_{s}\right\}$ , the target domain data $X_{t}$ , unknown labels of the target domain $Y_{t}$ (the initialization can be obtained by cluster algorithm), model parameter values of β,α,ρ,θ and the threshold of iteration stop ε , and the maximal iteration number Z . **Output:** The contribution matrix $\lambda_{k,c}$ matches each instance to the mean points of each class in the entire domain, the decision function W and the label matrix Y . **Procedure:** 1. Initialize the label values for unlabeled data from the target domain. 2. Compute the means of different classes in the target domain and the source domain respectively, denoted as $\mu_{t,c}$ and $\mu_{s,c}$ , c=0,1,2,…,C . 3. Then compute the mean of different class data in the overall domain (i.e., integrate the source domain and the target domain), denoted as $\mu_{c}=\frac{1}{2}\left(\mu_{s,c}+\mu_{t,c}\right)$ 4. Obtain the initialization $\lambda_{k,c}^{0}$ of $\lambda_{k,c}$ using (18); 5. Obtain the initialization $W^{0}$ of W using (21); 6. Obtain the initialization $Y^{0}$ of Y using (23); 7. Compute the value of the objective function $\Theta \left(\lambda_{k,c}^{0}{\mathrm{,W}}^{0}{\mathrm{,Y}}^{0}\right)$ ; 8.**for** *z* = 1**to** *Z***do**: { 8.1 Fix the current W and Y for updating $\lambda_{k,c}$ to $\lambda_{k,c}^{z}$ by Eq. (18)； 8.2 Fix the current $\lambda_{k,c}$ and Y for updating W to $W^{z}$ by Eq. (21)； 8.3 Fix the current $\lambda_{k,c}$ and W for updating Y to $Y^{z}$ by Eq. (23)； }**while** $\left|\Theta \left(\lambda_{k,c}^{z}{\mathrm{,W}}^{z}{\mathrm{,Y}}^{z}\right)-\Theta (\lambda_{k,c}^{z-1}{\mathrm{,,W}}^{z-1}{\mathrm{,,Y}}^{z-1})\right|\geq \varepsilon$ **9. return** $\lambda_{k,c}$ , W , and Y ;

### 4.2 Computational complexity

This article uses Big O to analyze the computational complexity of Algorithm 1. The proposed method C-PDDM mainly consists of two joint optimization parts: P-DDM and target label propagation. Specifically, we first construct the *k-Nearest Neighbor (*i.e.*, k-NN*) graph and compute the kernel matrix K in advance requiring computational costs of $O\left(dn^{2}\right)$ and $O\left(dN^{2}\right)$ , respectively. Then, the optimization process of Algorithm 1 requires T iterations to complete with the P-DDM minimization (including possibility membership inference) process requires $O\left(d^{3}+N^{2}+d^{2}N\right)$ . The target label matrix $F_{t}$ requires $O\left(3n^{3}+n^{2}c\right)$ to complete inferring thing. The target classification model W requires $O\left(nc^{2}+dc^{2}\right)$ to finish updating, Therefore, the overall computational cost of Algorithm 1 is $O\left(T\left(d^{3}+N^{2}+d^{2}N+3n^{3}+n^{2}c\right)+dn^{2}+dN^{2}\right)$ .

Before training in Algorithm 1, pre-computing the C-PDDM kernel matrix and Laplacian graph matrix and loading them into memory can further improve the computational efficiency of Algorithm 1. In short, the proposed algorithm is feasible and effective in practical applications.

## 5. Analysis and discussion of C-PDDM

### 5.1. Analysis of convergence

To prove the convergence of Algorithm 1, the following lemma is proposed.

**Lemma 1 (**Nie et al., 2010). For any two non-zero vectors $V_{1},V_{2}\in {\mathbb{R}}^{d}$ , the following inequality holds:

(24) $${\left\|V_{1}\right\|}_{2}-\frac{{\left\|V_{1}\right\|}_{2}^{2}}{2{\left\|V_{2}\right\|}_{2}^{}}\leq {\left\|V_{2}\right\|}_{2}^{}-\frac{{\left\|V_{2}\right\|}_{2}^{2}}{2{\left\|V_{2}\right\|}_{2}^{}}$$

Then, we prove the convergence of the proposed algorithm through Theorem 5.

**Theorem 5**. Algorithm 1 decreases the objective value of the optimization problem (17) in each iteration and converges to the optimal solution.

**Proof.** For expression simply, the updated results of optimization variables $\lambda_{k,c}$ , W , and Y after τ -th iteration are denoted as $\lambda_{k,c}^{\tau }$ , $W^{\tau }$ , and $Y^{\tau }$ , respectively. The internal loop iteration update in Step 8 of Algorithm 1 corresponds to the following optimization problem:

(25) $$\begin{array}{l} \Theta \left(\lambda_{k},Y,W\right)\\ =\underset{\lambda_{k,c},Y,W}{\min}\sum_{c=0}^{C}\sum_{k=1}^{N}\lambda_{k,c}^{2}{\left\|\phi \left(x_{k,c}^{}\right)-\mu_{c}^{}\right\|}_{H}^{2}\\ +\beta \sum_{c=0}^{C}\sum_{k=1}^{N}\left(\lambda_{k,c}^{2}\ln\lambda_{k,c}^{2}-\lambda_{k,c}^{2}\right)+\alpha tr(Y^{T}LY)\\ +\sum_{c=0}^{C}\sum_{k=1}^{N}\lambda_{k,c}^{2}{\left\|\phi^{T}(x_{k})W-y_{k}\right\|}_{H}^{2}+\rho tr\left(W^{T}UW\right) \end{array}$$

According to the definition of matrix U , we have:

(26) $$\begin{array}{l} Ζ\left(\tau +1\right)+\rho \sum_{i=1}^{N}\frac{{\left\|W{\left(i,:\right)}^{\tau +1}\right\|}_{2}^{2}}{{\left\|W{\left(i,:\right)}^{\tau }\right\|}_{2}}\\ \leq Ζ\left(\tau \right)+\rho \sum_{i=1}^{N}\frac{{\left\|W{\left(i,:\right)}^{\tau }\right\|}_{2}^{2}}{{\left\|W{\left(i,:\right)}^{\tau }\right\|}_{2}} \end{array}$$

where

$$\begin{array}{l} Ζ\left(e\right)=\sum_{c=0}^{C}{\left(\lambda_{}^{{\left(c\right)}^{e}}\right)}^{2}{\left\|\phi \left(X_{}^{\left(c\right)}\right)-\mu_{}^{\left(c\right)}\right\|}_{H}^{2}\\ +\beta \sum_{c=0}^{C}\left({\left(\lambda_{}^{{\left(c\right)}^{e}}\right)}^{2}\ln{\left(\lambda_{}^{{\left(c\right)}^{e}}\right)}^{2}-{\left(\lambda_{}^{{\left(c\right)}^{e}}\right)}^{2}\right)\\ +\alpha tr\left({\left(Y^{e}\right)}^{T}LY^{e}\right)\\ +\sum_{c=0}^{C}{\left(\lambda_{}^{{\left(c\right)}^{e}}\right)}^{2}{\left\|\phi^{T}\left(X^{\left(c\right)}\right)W^{e}-Y^{e}\right\|}_{H}^{2} \end{array}$$

Based on Lemma 1, we can obtain the following inequality:

(27) $$\begin{array}{l} \sum_{j=1}^{N}\left({\left\|{\left(W\right)}_{j,:}^{\tau +1}\right\|}_{2}-\frac{{\left\|{\left(W\right)}_{j,:}^{\tau +1}\right\|}_{2}^{2}}{2{\left\|{\left(W\right)}_{j,:}^{\tau }\right\|}_{2}^{}}\right)\\ \leq \sum_{j=1}^{N}\left({\left\|{\left(W\right)}_{j,:}^{\tau }\right\|}_{2}^{}-\frac{{\left\|{\left(W\right)}_{j,:}^{\tau }\right\|}_{2}^{2}}{2{\left\|{\left(W\right)}_{j,:}^{\tau }\right\|}_{2}^{}}\right) \end{array}$$

Therefore, we can derive:

(28) $$\begin{array}{l} Ζ\left(\tau +1\right)+\rho \sum_{i=1}^{N}{\left\|W_{j,:}^{\tau +1}\right\|}_{2}\\ \leq Ζ\left(\tau \right)+\rho \sum_{i=1}^{N}{\left\|W_{j,:}^{\tau }\right\|}_{2} \end{array}$$

Finally, Theorem 6 is proved.

According to the update rule in Algorithm 1 and Theorem 6, it is known that the optimization objective (17) is a decreasing function concerning the objective value. Therefore, it can be inferred that Algorithm 1 can effectively converge to the optimal solution.

### 5.2. Analysis of generalization

Rademacher complexity can effectively measure the ability of a function set to fit noise (Ghifary et al., 2017; Tao and Dan, 2021). Therefore, we will derive the generalization error bound of the proposed method through Rademacher complexity. Let H:={h:→} be a set of hypothesis functions in the RKHS H space, where X is a compact set and Y is a label space. Given a loss function $loss\left(\cdot ,\cdot \right):Y\times Y\to {\mathbb{R}}_{+}$ and a.

neighborhood distribution D on X , the expected loss of two hypothesis functions $h,\tilde{h}\in H$ is defined as:

$$ℒ(h,\tilde{h})=E_{x∼}[loss\left(h(x),\tilde{h}(x)\right)]$$

The domain distribution difference between the source domain distribution ℙ and the target domain distribution ℚ can be defined as:

(29) $$disc(\mathbb{P},\mathbb{Q})=\underset{h,\tilde{h}\in H}{\sup}\{{ℒ}_{\mathbb{P}}\left(h,\tilde{h}\right)-{ℒ}_{\mathbb{Q}}\left(h,\tilde{h}\right)\}$$

Let $f_{\mathbb{P}}$ and $f_{\mathbb{Q}}$ be the true label functions for ℙ and ℚ , respectively, and let the corresponding optimized hypothesis functions be:

$$\begin{array}{l} h_{\mathbb{P}}^{*}:=\underset{h\in H}{argmin}{ℒ}_{\mathbb{P}}\left(h,f_{\mathbb{P}}\right)\\ h_{\mathbb{Q}}^{*}:=\underset{h\in H}{argmin}{ℒ}_{\mathbb{Q}}\left(h,f_{\mathbb{Q}}\right) \end{array}$$

Their corresponding expected loss is denoted as $L_{\mathbb{P}}\left(h_{\mathbb{Q}}^{*},h_{\mathbb{P}}^{*}\right)$ . Our C-PDDM method achieves the empirical loss target of $L_{\mathbb{P}}\left(h_{\mathbb{Q}}^{*},h_{\mathbb{P}}^{*}\right)$ through the objective function R(Y,W) .

The following theorem gives the generalization error bound of the proposed method:

**Theorem 6 (Generalization Error Bound)** (Nie et al., 2010). Let $H:=\{f\in ℋ:\to \mathbb{R},{\left\|f\right\|}_{ℋ}\leq 1and{\left\|f\right\|}_{\infty }\leq r\}$ is a function set of RKHS H . $X_{X}^{\mathbb{P}}=\left(x_{1}^{s},\ldots ,x_{n_{s}}^{s}\right)∼\mathbb{P}$ and $X_{X}^{\mathbb{Q}}=\left(x_{1}^{t},\ldots ,x_{n_{t}}^{t}\right)∼\mathbb{Q}$ are datasets of the source domain and the target domain, respectively. q-Lipschitz function loss is loss(⋅,⋅):Y×Y→[0,q] . When a,b∈Y×Y , |loss(a)−loss(b)|=q|a−b| . The generalization error bound for any hypothesis function h∈H with a probability of at least 1−δ of having Rademacher complexity ${ℜ}_{X_{X}^{\mathbb{P}}}\left(H\right)$ on $X_{X}^{\mathbb{P}}$ is:

(30) $$\begin{array}{l} L_{\mathbb{Q}}\left(h,f_{\mathbb{Q}}\right)-L_{\mathbb{Q}}\left(h_{\mathbb{Q}}^{*},f_{\mathbb{Q}}\right)\leq L_{\mathbb{P}}\left(h,h_{\mathbb{P}}^{*}\right)+2q{ℜ}_{X_{X}^{\mathbb{P}}}\left(H\right)\\ +3q\sqrt{\frac{\log\frac{2}{\delta }}{2N}}+8q\sqrt{\Omega \left(\lambda_{k},X_{s},X_{t}\right)}+R\left(Y,W\right) \end{array},$$

where ${ℜ}_{X_{X}^{\mathbb{P}}}\left(H\right)$ is Rademacher complexity.

Theorem 6 shows that the possibilistic distribution distance measure $\Omega \left(\lambda_{k},X_{s},X_{t}\right)$ and the model alignment function R(Y,W) can simultaneously control the generalization error bound of the proposed method. Therefore, the proposed method can effectively improve its generalization performance in domain adaptation by minimizing both the possibilistic distribution distance between domains and model bias. The experimental results on real-world datasets also confirm this conclusion.

### 5.3. Discussion of kernel selection

The literature (32) theoretically analyzed and pointed out that the Gaussian kernel cluster provides an effective RKHS embedding space for the consistency estimation of domain distribution distance measure. The detailed derivation process can be found in Sriperumbudur et al. (2010a,b). Therefore, all the kernel functions used in this paper are Gaussian kernel $k_{\sigma }=e^{-x_{i}-x_{j}{}_{2}^{2}/2\sigma^{2}}$ . In order to illustrate the impact of the Gaussian kernel bandwidth on the distribution of sample RKHS embedding, the following theorem is introduced:

**Theorem 7** (Sriperumbudur et al., 2010a). The function set of Gaussian kernel.

(31) $$\begin{array}{l} K_{s}=\{k_{\sigma }=e^{-{\left\|x_{i}-x_{j}\right\|}_{2}^{2}/2\sigma^{2}},x_{i},x_{j}\in {\mathbb{R}}^{d},\\ {}_{}{}_{}{}_{}\sigma \in [\sigma_{0},\infty ),\sigma_{0}>0\} \end{array}$$

For any $k_{\sigma },k_{\theta }\in K_{s}$ and 0<θ<σ<∞ , then $\zeta_{k\sigma }\left(X^{s},X^{t}\right)\geq \zeta_{k\theta }\left(X^{s},X^{t}\right)$ .

According to Theorem 7, the larger the kernel bandwidth, the larger the RKHS embedding distance of the domain distribution, which slows down the convergence speed of the domain distribution distance measure $\Omega \left(\lambda_{k},X_{s},X_{t}\right)$ based on the soft clustering hypothesis of the MMD criterion. In order to further study the performance impact of Gaussian kernel bandwidth, the Gaussian kernel bandwidth is parameterized, that is, the generalized Gaussian kernel function is defined as:

(32) $$k_{\sigma /\theta }\left(x,X_{i}\right)=\exp\left(-{\left\|x-X_{i}\right\|}_{2}^{2}/2{\left(\sigma /\theta \right)}^{2}\right)$$

where θ is a tunable parameter, as will be shown in the experimental analysis below. When θ is too large, the samples within the domain are highly cohesive, leading to a certain degree of mixing between positive and negative classes, which is not conducive to effective classification of the model. Conversely, when θ is too small, it may slow down the convergence of the distribution distance measurement algorithm based on the possibilistic clustering hypothesis to some extent. Therefore, this paper limits $\theta \in \left[1,\theta_{0}\right]$ , where $\theta_{0}$ is a sufficiently large tunable parameter. The above analysis shows that the distribution distance measurement based on the possibilistic clustering hypothesis can not only constrain the divergence of the distributions between domains to be as consistent as possible, but also reduce the divergence of the sample distributions within each domain within a certain range of kernel bandwidths, thereby accelerating the convergence speed of the domain distribution divergence difference measurement and further improving the execution efficiency of the algorithm.

It is worth noting that kernel selection is an open problem in kernel learning methods. Recently, some studies have proposed the use of Multi-Kernel Learning (MKL) (Long et al., 2015) to overcome the kernel selection problem in single-kernel learning methods. Therefore, we can also use MKL to improve the performance of the proposed method. Specifically, the first step is to construct a new space that spans multiple kernel feature mappings, represented by ${\left\{\phi_{a}\right\}}_{a=1}^{℧}$ , which projects X into ℧ different spaces. Then, an orthogonal integration space can be built by connecting these ℧ spaces, and $\tilde{\phi }\left(x_{i}\right)={\left[\phi_{1}{\left(x_{i}\right)}^{T},\phi_{2}{\left(x_{i}\right)}^{T},\ldots ,\phi_{℧}{\left(x_{i}\right)}^{T}\right]}^{T}\in {\mathbb{R}}^{℧N}$ represents the mapping features in the final space, where $x_{i}\in X$ . In addition, the kernel matrix in this final space can be written as $K_{new}=\left[\tilde{K_{1}};\tilde{K_{2}};...;\tilde{K_{℧}}\right]$ , where $\tilde{K_{i}}$ is the *i*-th kernel matrix from ℧ feature spaces. The kernel functions that can be used in practice include the Gaussian kernel function, inverse square distance kernel function $K_{ij}=1/\left(1+\sigma {\left\|x_{i}-x_{j}\right\|}^{2}\right)$ , Laplacian kernel function $K_{ij}=\exp\left(-\sqrt{\sigma }\left\|x_{i}-x_{j}\right\|\right)$ , and inverse distance kernel function $K_{ij}=1/\left(1+\sqrt{\sigma }\left\|x_{i}-x_{j}\right\|\right)$ , etc.

## 6. Experiments

### 6.1. Emotional databases and data preprocessing

In order to make a fair comparison with stat-of-the-art (SOTA) methods, a large number of experiments were conducted for effective validation on two well-known open datasets [i.e., SEED (Zheng and Lu, 2015) and SEED-IV (Zheng et al., 2019)]. The SEED dataset has a total of 15 subjects participating in the experiment to collect data, each subject needs to have three sessions at different times, each session contains 15 trials, with a total of 3 emotional stimuli (negative, neutral, and positive). In the SEED-IV dataset, there are also 15 subjects participating in the experiment to collect data, each subject needs to have three sessions at different times, each session contains 24 trials, with a total of 4 emotional stimuli (happy, sad, fearful, and peaceful).

The EEG signals of the two datasets (i.e., SEED and SEED-IV) are collected simultaneously from the 62-channel ESI Neuroscan system. In the EEG signal preprocessing, the down-sampled data sampling rate is reduced to 200 Hz, then the environmental noise data is manually removed, and the data is filtered through a 0.3 Hz–50 Hz band-pass filter. In each trial, the data is divided into multiple segments with a length of 1 s. Based on the predefined 5 frequency band-passes [Delta (1–3 Hz), Theta (4–7 Hz), Alpha (8–13 Hz), Beta (14–30 Hz), and Gamma (31–50 Hz)], the corresponding differential entropy (DE) is extracted to represent the logarithmic power spectrum in the specified frequency band-pass, and a total of 310 features (5 frequency bands and 62 channels) are obtained in each EEG segment. Then, all features are smoothed by the Linear Dynamic System (LDS) method, which can utilize the time dependency of emotion transitions and filter out the noise EEG components unrelated to emotions (Shi and Lu, 2010).

#### 6.1.1. Settings

The settings of the hyper-parameter for the C-PDDM method are also crucial before analyzing the experimental evaluation results. For all methods, in both the source and target domains, a Gaussian kernel $K(x,x_{i})=\exp(-{\left\|x-x_{i}\right\|}^{2}/2\sigma^{2})$ is used, where σ can be obtained by minimizing MMD to obtain a benchmark test. Based on experience, we first select σ as the square root of the average norm of the binary training data, and $\sigma \sqrt{C}$ (where C is the number of classes) for multi-class classification. The underlying geometric structure depends on *k* neighbors to compute the Laplacian matrix. In the experiment of this paper, it can be observed that the performance slightly varies when *k* is not large. Therefore, to construct the nearest neighbor graph in C-PDDM, this paper conducts a grid search for the optimal number of nearest *k* neighbors in {3,5,10,15,17} , and provides the best recognition accuracy results from the optimal parameter configuration.

Before presenting the detailed evaluation, it is necessary to explain how the hyper-parameters of C-PDDM are tuned. Based on experience, the parameter β is used to balance the fuzzy entropy and domain probability distribution alignment in the objective function (16). Both parameters α and ρ are adjustable parameters, and they are used to balance the importance of structure description and feature selection. Therefore, these two parameters have a significant impact on the final performance of the method.

Considering that parameter uncertainty is still an open problem in the field of machine learning, we determine these parameters based on previous work experience. Therefore, we evaluate all methods on the dataset by empirically searching the parameter space to obtain the optimal parameter settings and give the best results for each method. Except for special cases, all parameters of all relevant methods are tuned to obtain the optimal results.

As unsupervised domain adaptation does not have target labels to guide standard cross-validation, we perform leave-one-subject-out on the two datasets: SEED and SEED-IV (the details of this protocol are shown in Section 6.2). We obtain the optimal parameter values on { $10^{-6}$ , $10^{-5}$ , …, $10^{5}$ , $10^{6}$ } by obtaining the highest average accuracy on the two datasets using the above method. This strategy often constructs a good C-PDDM model for unsupervised domain adaptation, and a similar strategy is adopted to find the optimal parameter values for other domain adaptation methods. In the following sub-sections, a set of experiments is set up to test the sensitivity of the proposed method C-PDDM to parameter selection (i.e., Section 6.4.1), in order to verify that C-PDDM can achieve stable performance within a wide range of parameter values. In addition, the hyper-parameters of other methods are selected according to the original literature.

### 6.2. Experiment protocols

In order to fully verify the robustness and stability of the proposed method, we adopt four different validation protocols (leave-one-subject-out) (Zhang et al., 2021) to compare the proposed method with the SOTA methods.

1. **Cross-subject cross-session leave-one-subject-out cross-validation.** To fully estimate the robustness of the model on unknown subjects and trials, this paper uses a strict leave-one-out method cross-subject cross-session to evaluate the model. All session data of one subject is used as the target domain, and all sessions of the remaining subjects are used as the source domain. We repeat the training and validation until all sessions of each subject have been used as the target domain once. Due to the differences between subjects and sessions, this evaluation protocol poses a significant challenge to the effectiveness of models in emotion recognition tasks based on EEG.
2. **Cross-subject single-session leave-one-subject-out cross-validation.** This is the most widely used validation scheme in emotion recognition tasks based on EEG (Luo et al., 2018; Li J. et al., 2020). One session data of a subject is treated as the target domain, while the remaining subjects are treated as the source domain. We repeat the training and validation process until each subject serves as the target once. As with other studies, we only consider the first session in this type of cross-validation.
3. **Within-subject cross-session leave-one-session-out cross-validation.** Similar to existing methods, a time series cross-validation method is employed here, where past data is used to predict current or future data. For a subject, the first two sessions are treated as the source domain, and the latter session is treated as the target domain. The average accuracy and standard deviation across subjects are calculated as the final results.
4. **Within-subject single-session cross-validation.** Following the validation protocols proposed in existing studies (Zheng and Lu, 2015; Zheng et al., 2019), for each session of a subject, we take the first 9 (SEED) or 16 (SEED-IV) trials as the source domain and the remaining 6 (SEED) or 8 (SEED-IV) trials as the target domain. The results are reported as the average performance of all participants. In the performance comparison of the following four different validation protocols, we use “*” to indicate the replicated model results.

### 6.3. Results analysis on SEED and SEED-IV

#### 6.3.1. Cross-subject cross-session

For verifying the efficiency and stability of the model under cross-subject and cross-session conditions, we used cross-subject cross-session leave-one-subject-out cross-validation on the SEED and SEED-IV databases to validate the proposed C-PDDM. As shown in Tables 1, 2, the results show that our proposed model achieved the highest accuracy of emotion recognition. The C-PDDM method, with or without using deep features, achieved emotion recognition performances of 73.82 ± 6.12 and 86.49 ± 5.20 for the three-class classification task on SEED, and 67.83 ± 8.06 and 72.88 ± 6.02 for the four-class classification task on SEED-IV. Compared with existing research, the proposed C-PDDM has a slightly lower accuracy on SEED-IV than PR-PL, but PR-PL uses adversarial learning, which has a higher computational cost. In addition, the proposed C-PDDM method has the best recognition performance in the other three cases. These results indicate that the proposed C-PDDM has a higher recognition accuracy and better generalization ability, and is more effective in emotion recognition.

**Table 1:** The mean accuracies (%) and standard deviations (%) of emotion recognition on the SEED database using cross-subject cross-session leave-one-subject-out cross-validation.

Methods	Pacc	Methods	Pacc
**Traditional machine learning methods**			
RF (Breiman, 2001)	69.60 ± 7.64	KNN (Coomans and Massart, 1982)	60.66 ± 7.93
SVM* (Suykens and Vandewalle, 1999)	62.24 ± 5.48	Adaboost (Zhu et al., 2006)	71.87 ± 5.70
TCA* (Pan et al., 2011)	65.31 ± 6.04	CORAL (Sun et al., 2016)	69.22 ± 4.11
SA (Li Y. et al., 2020)	61.41 ± 9.75	GFK* (Gong et al., 2012)	67.36 ± 6.52
DICE* (Liang et al., 2018)	73.56 ± 4.23	C-PDDM	**73.82 ± 6.12**
**Deep learning methods**			
DCORAL* (Sun et al., 2016)	80.87 ± 6.04	DAN* (Long et al., 2015)	82.51 ± 3.71
DDC (Tzeng et al., 2014)	82.17 ± 4.96	DANN* (Ganin et al., 2016)	84.79 ± 6.44
PR-PL (Zhou et al., 2022)	85.56 ± 4.78	C-PDDM+ResNet101	**86.49 ± 5.20**

Here, the model results reproduced by us are indicated by “*”. The bold values are the best performance in tables.

**Table 2:** The mean accuracies (%) and standard deviations (%) of emotion recognition on SEED-IV database using cross-subject cross-session leave-one-subject-out cross-validation.

Methods	Pacc	Methods	Pacc
**Traditional machine learning methods**			
RF	50.98 ± 9.20	KNN	40.83 ± 7.28
SVM	51.78 ± 12.85	Adaboost	53.44 ± 9.12
TCA	56.56 ± 13.77	CORAL	49.44 ± 9.09
SA	64.44 ± 9.46	GFK	45.89 ± 8.27
KPCA (Suykens and Vandewalle, 1999)	51.76 ± 12.89	DNN (Suykens and Vandewalle, 1999)	49.35 ± 9.74
DICE	66.75 ± 7.25	C-PDDM	**67.83 ± 8.06**
**Deep learning methods**			
DGCNN (Song et al., 2018)	52.82 ± 9.23	DAN	58.87 ± 8.13
RGNN (Zhong et al., 2020)	73.84 ± 8.02	BiHDM (Li Y. et al., 2020)	69.03 ± 8.66
BiDANN (Li et al., 2018c)	65.59 ± 10.39	DANN	54.63 ± 8.03
PR-PL	**74.92 ± 7.92**	C-PDDM+ResNet101	72.88 ± 6.02

The bold values are the best performance in tables.

#### 6.3.2. Cross-subject single-session

Table 3 summarizes the model results of the recognition task under cross-subject single-session leave-one-subject-out and compares them with the performance of the latest methods in the literature. All results are presented in the form of mean ± standard deviation. The results show that our proposed model (C-PDDM) achieves the best performance (74.92%) with a standard deviation of 8.16 when compared with traditional machine learning methods. The recognition performance of C-PDDM is better than the DICE method, indicating that the C-PDDM method is superior to the DICE method in dealing with noisy situations. When compared with the latest deep learning methods, especially with deep transfer learning networks based on DANN (Li J. et al., 2020) [such as ATDD-DANN (Du et al., 2020), R2GSTNN(Li et al., 2019), BiHDM (Li Y. et al., 2020), BiDANN (Li et al., 2018c), WGAN-GP (Luo et al., 2018)], the proposed C-PDDM method effectively addresses individual differences and noisy label issues in aBCI applications. The recognition performance of PR-PL is slightly better than the C-PDDM, which may be because the PR-PL method uses adversarial loss for model learning, resulting in higher computational costs. Overall, the C-PDDM method has a competitive result, indicating that the C-PDDM method has better generalization performance in cross-subject within the same session.

**Table 3:** The mean accuracies (%) and standard deviations (%) of emotion recognition on the SEED database using cross-subject single-session leave-one-subject-out cross-validation.

Methods	Pacc	Methods	Pacc
**Traditional machine learning methods**			
TKL (Li et al., 2018c)	63.54 ± 15.47	T-SVM* (Li et al., 2018c)	68.57 ± 9.54
TCA	63.64 ± 14.88	TPT* (Suykens and Vandewalle, 1999)	73.86 ± 11.05
KPCA	61.28 ± 14.62	GFK	71.31 ± 14.09
SA*	66.00 ± 10.89	DICA (Ma et al., 2019)	69.40 ± 07.80
DNN	61.01 ± 12.38	SVM	58.18 ± 13.85
DICE	74.22 ± 7.33	C-PDDM	**74.92 ± 8.16**
**Deep learning methods**			
DGCNN	79.95 ± 9.02	DAN	83.81 ± 8.56
RGNN	85.30 ± 6.72	BiHDM	85.40 ± 7.53
WGAN-GP (Luo et al., 2018)	87.10 ± 7.10	MMD (Li J. et al., 2020)	80.88 ± 10.10
ATDD-DANN (Du et al., 2020)	90.92 ± 1.05	JDA-Net (Li J. et al., 2020)	88.28 ± 11.44
R2G-STNN (Li et al., 2019)	84.16 ± 7.63	SimNet* (Pinheiro, 2018)	81.58 ± 5.11
BiDANN	83.28 ± 9.60	DResNet (Ma et al., 2019)	85.30 ± 8.00
ADA (Li J. et al., 2020)	84.47 ± 10.65	DANN	81.65 ± 9.92
PR-PL	**93.06 ± 5.12**	C-PDDM+ResNet101	92.19 ± 4.70

Here, the model results reproduced by us are indicated by “*”. The bold values are the best performance in tables.

#### 6.3.3. Within-subject cross-session

By calculating the mean and standard deviation of the experimental results for each subject, the cross-session cross-validation results for each subject on the different datasets SEED and SEED-IV are shown in Tables 4, 5, respectively. For these two datasets, our proposed C-PDDM method, which compared with the existing traditional machine learning methods, has results close to or better than the DICE method on both SEED and SEED-IV. This may be because each subject is less likely to generate noisy data in different sessions, which does not highlight the advantages of C-PDDM. In addition, for the SEED-IV dataset (four-class emotion recognition), regardless of traditional machine learning or the latest deep learning methods, the performance of the C-PDDM method is the best when the number of categories increases. This indicates that the proposed method is more accurate and has stronger scalability in more nuanced emotion recognition tasks.

**Table 4:** The mean accuracies (%) and standard deviations (%) of emotion recognition on the SEED database using within-subject cross-session cross-validation.

Methods	Pacc	Methods	Pacc
**Traditional machine learning methods**			
RF	76.42 ± 11.15	KNN*	72.96 ± 12.10
TCA*	77.63 ± 11.49	CORAL	84.18 ± 9.81
SA*	67.79 ± 7.43	GFK*	79.28 ± 7.44
DICE	**81.58 ± 7.55**	C-PDDM	**81.58 ± 9.30**
**Deep learning methods**			
DAN	89.16 ± 7.90	SimNet	86.88 ± 7.83
DDC	91.14 ± 5.61	ADA	89.13 ± 7.13
DANN	89.45 ± 6.74	MMD	84.38 ± 12.05
JDA-Net	91.17 ± 8.11	DCORAL (Sun et al., 2016)	88.67 ± 6.25
PR-PL	**93.18 ± 6.55**	C-PDDM+ResNet101	92.56 ± 5.29

The bold values are the best performance in tables.

**Table 5:** The mean accuracies (%) and standard deviations (%) of emotion recognition on SEED-IV database using within-subject cross-session cross-validation.

Methods	Pacc	Methods	Pacc
**Traditional machine learning methods**			
RF	60.27 ± 16.36	KNN	54.18 ± 16.28
TCA*	59.49 ± 12.07	CORAL*	66.88 ± 14.67
SA*	56.94 ± 11.45	GFK*	60.66 ± 10.00
DICE	69.68 ± 12.52	C-PDDM	**70.48 ± 9.08**
**Deep learning methods**			
DCORAL (Chen et al., 2021)	65.10 ± 13.20	DAN	60.20 ± 10.20
DDC (Chen et al., 2021)	68.80 ± 16.60	MEERNet (Chen et al., 2021)	72.10 ± 14.10
PR-PL	74.62 ± 14.15	C-PDDM+ResNet101	**76.29 ± 11.36**

The bold values are the best performance in tables.

#### 6.3.4. Within-subject single-session

The previous evaluation strategy only considered the first two sessions of the SEED dataset as the source domain for the experiment. The evaluation results of emotion recognition for each subject within each session are presented in Table 6. When compared with traditional machine learning methods, the C-PDDM method has comparable performance, and it still outperforms the performance of the DICE method. When compared with the latest deep learning methods, the C-PDDM method achieves the highest recognition performance, reaching 96.38%, which is even higher than the PR-PL method. This comparison demonstrates the high efficiency and reliability of the proposed C-PDDM method in various emotion recognition applications.

**Table 6:** The mean accuracies (%) and standard deviations (%) of emotion recognition on the SEED database using within-subject single-session cross-validation.

Methods	Pacc	Methods	Pacc
**Traditional machine learning methods**			
SVM*	77.80 ± 12.61	GRSLR (Li et al., 2018a)	**87.39 ± 8.64**
RF	78.46 ± 11.77	GSCCA (Zheng, 2017)	82.96 ± 9.95
CCA	77.63 ± 13.21	DBN (Zheng et al., 2015)	86.08 ± 8.34
DICE	86.28 ± 9.22	C-PDDM	86.74 ± 7.59
**Deep learning methods**			
DGCNN	90.40 ± 8.49	RGNN	94.24 ± 5.95
ATDD-DANN	91.08 ± 6.43	BiHDM	93.12 ± 6.06
R2G-STNN	93.38 ± 5.96	SimNet*	90.13 ± 10.84
BiDANN	92.38 ± 7.04	STRNN (Zhang et al., 2019a)	89.50 ± 7.63
GCNN (Breiman, 2001)	87.40 ± 9.20	DANN	91.36 ± 8.30
PR-PL	94.84 ± 9.16	C-PDDM+ResNet101	**96.38 ± 6.88**

The bold values are the best performance in tables.

For the SEED-IV dataset, we calculated the performance of all three sessions (emotional categories: happiness, sadness, fear, and neutral). Our proposed model outperforms the existing latest classical research methods and achieves the highest accuracy of 71.85 and 83.94% in Table 7. This comparison shows that the more emotional categories there are, the more prominent the generalization of the proposed C-PDDM method in applications.

**Table 7:** The mean accuracies (%) and standard deviations (%) of emotion recognition on SEED-IV database using within-subject single-session cross-validation.

Methods	Pacc	Methods	Pacc
**Traditional machine learning methods**			
SVM	56.61 ± 20.05	GRSLR	69.32 ± 19.57
RF	50.97 ± 16.22	GSCCA	69.08 ± 16.66
CCA	54.47 ± 18.48	DBN	66.77 ± 07.38
DICE	71.67 ± 11.29	C-PDDM	**71.85 ± 9.18**
**Deep learning methods**			
DGCNN	69.88 ± 16.29	RGNN	79.37 ± 10.54
GCNN	68.34 ± 15.42	BiHDM	74.35 ± 14.09
A-LSTM (Breiman, 2001)	69.50 ± 15.45	SimNet*	71.38 ± 13.12
BiDANN	70.29 ± 12.63	DANN	63.07 ± 12.66
PR-PL	83.33 ± 10.61	C-PDDM+ResNet101	**83.94 ± 11.39**

The bold values are the best performance in tables.

### 6.4. Discussion

For comprehensively study the performance of the model, we evaluated the effects of different settings in C-PDDM. Please note that all the results presented in this section are based on the SEED dataset, using the cross-subject single-session cross-validation evaluation protocol.

#### 6.4.1. Ablation study

We conducted ablation studies to systematically explore the effectiveness of different components in the proposed C-PDDM model and their respective contributions to the overall performance of the model. As shown in Table 8, when 5 labeled samples existed at each category in the target domain, the recognition accuracy (93.83% ± 5.17) is very close to the recognition accuracy of C-PDDM (unsupervised learning) (92.19% ± 4.70). This decrease indicates the impact of individual differences on model performance and highlights the huge potential of transfer learning in aBCI applications. Moreover, the results show that simultaneously preserving the local structure of data in both the source and target domains helps improve model performance; otherwise, the recognition accuracy decreases significantly (90.60% ± 5.29 and 91.37% ± 5.82, respectively). When ${\left\|W\right\|}_{2,1}$ is changed to ${\left\|W\right\|}_{2}$ , the model’s recognition accuracy drops to 91.84% ± 6.33. This result reflects the sample selection and denoising effects achieved when using $l_{2,1}$ constraint.

**Table 8:** The ablation study of our proposed model.

Ablation study about training strategy	Pacc
target prior information (5 labeled samples per category)	93.83 ± 5.17
only preserving the local structures on the source	90.60 ± 5.29
only preserving the local structures on the target	91.37 ± 5.82
imposing $l_{2}$ -norm on W	91.84 ± 6.33
fixed pseudo-labeling	89.95 ± 5.61
dynamic pseudo-labeling	92.19 ± 4.75
multiple kernel leaning	93.68 ± 6.04
**Hyper-parameter controlling strategy**	
α=0 (ignoring the local structures)	90.27 ± 5.51
fixed α=1 for local preserving regularization	91.93 ± 5.44
fixed β=100 for fuzzy entropy regularization	92.17 ± 6.30
fixed ρ for W regularization	92.16 ± 5.38
δ=0	88.47 ± 6.00
δ=0.3	88.91 ± 3.49
δ=0.5	92.19 ± 4.70
δ=0.85	91.83 ± 2.80
δ=1	89.85 ± 5.66
β=0 (ignoring the fuzzy entropy regularization)	90.56 ± 6.59
**The proposed model**	
C-PDDM+ResNet101	92.19 ± 4.70

For the pseudo-labeling method, when the pseudo-labeling method changes from fixed to linear dynamic, the corresponding accuracy increases from 89.95 to 92.19%. When adopting multi-kernel learning, the accuracy further improves to 93.68%. The results indicate that multi-kernel learning helps rationalize the importance of each kernel in different scenarios and enhances the generalization of the model.

Next, we analyze the impact of different hyper-parameters on the overall performance of the model. According to the experimental results, it can be seen that the recognition accuracy with α , β , ρ are dynamically learned better than fixed values. When ignoring the local structural information and fuzzy entropy information in the domain, the performance drops by about 2% (i.e., α=0 , α=1 , β=0 , and β=100 ). In addition, from the results, it can be inferred that the performance is optimal when the value of δ is around 0.5, indicating that the means of different categories in the source domain and target domain are equally important.

#### 6.4.2. Effect of noisy labels

**Figure 2:**
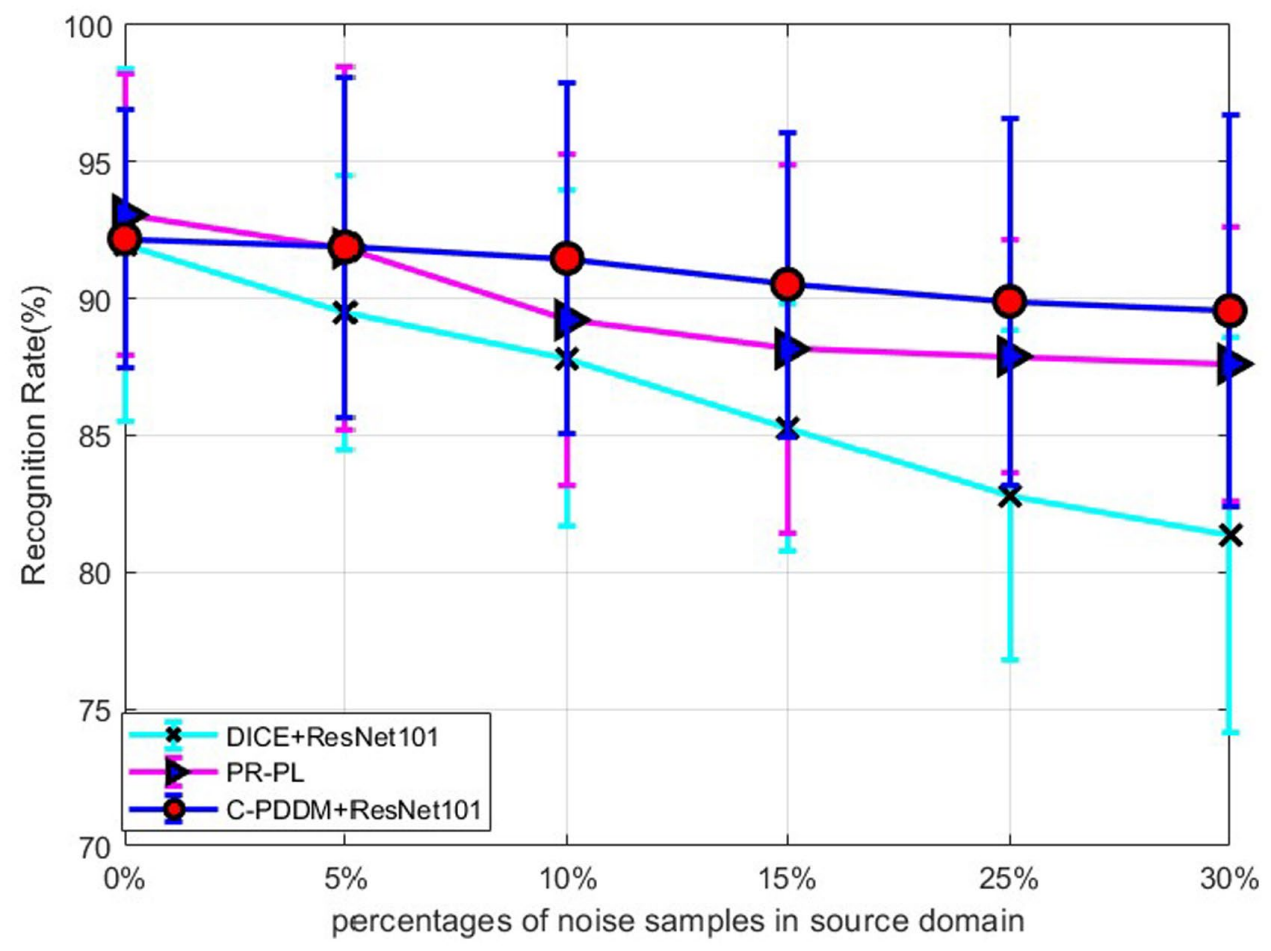
Robustness on source domain with different noise levels.

In order to further verify the robustness of the model in the noisy label learning process, we randomly add noise to the source labels at different ratios and test the performance of the corresponding model on unknown target data. Specifically, we replace the corresponding proportion of real labels in $Y^{s}$ with randomly generated labels to train the model by semi-supervised learning and then test the performance of the trained model in the target domain. It should be noted that only noise data is added in the source domain, and the target domain needs to be used for model evaluation. In the implementation, the noise ratios are adjusted to 5, 15, 25, and 30% of the sample number of the source domain, respectively. The results in Figure 2 show that the accuracy of the proposed C-PDDM decreases at the slowest rate as the number of noise increases. It indicates that C-PDDM is a reliable model with a high tolerance to noisy data. In future work, we can combine recently proposed new methods, such as Xiao et al. (2020) and (Jin et al. (2021), to further eliminate more common noise in EEG signals and improve the stability of the model in cross-corpus applications.

#### 6.4.3. Confusion matrices

**Figure 3:**
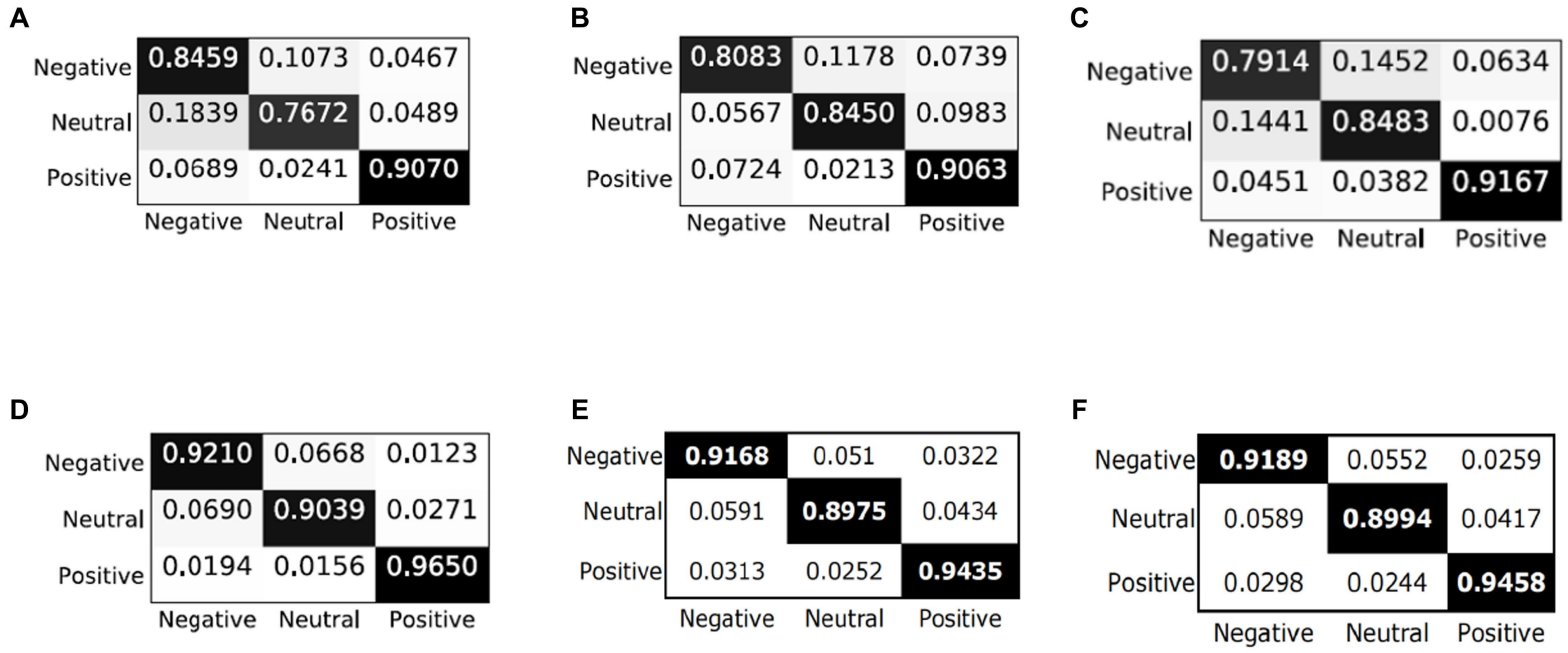
Confusion matrices of different models: **(A)** BiDANN; **(B)** BiHDM; **(C)** RGNN; **(D)** PR-PL; **(E)** DICE+ResNet101; and **(F)** C-PDDM+ResNet101.

In order to qualitatively study the performance of the model in each emotion category, we analyze the confusion matrix through visualization and compare the results with the latest models (i.e., BiDANN, BiHDM, RGNN, PR-PL, DICE ResNet101). As shown in Figure 3, all models are good at distinguishing positive emotions from other emotions (with recognition rates above 90%), but relatively not good at distinguishing negative emotions and neutral emotions. For example, the emotion recognition rate in BiDANN (Li et al., 2018c) is even lower than 80% (76.72%). In addition, the PR-PL method achieves the best performance, possibly due to its adoption of adversarial networks, but at the cost of increased computational expenses. Compared with other existing methods (Figures 3A–C,E), our proposed model can improve the model’s recognition ability, especially in distinguishing neutral and negative emotions, and its overall performance is better than the DICE method (as shown in Figures 3E,F).

#### 6.4.4. Convergence

**Figure 4:**
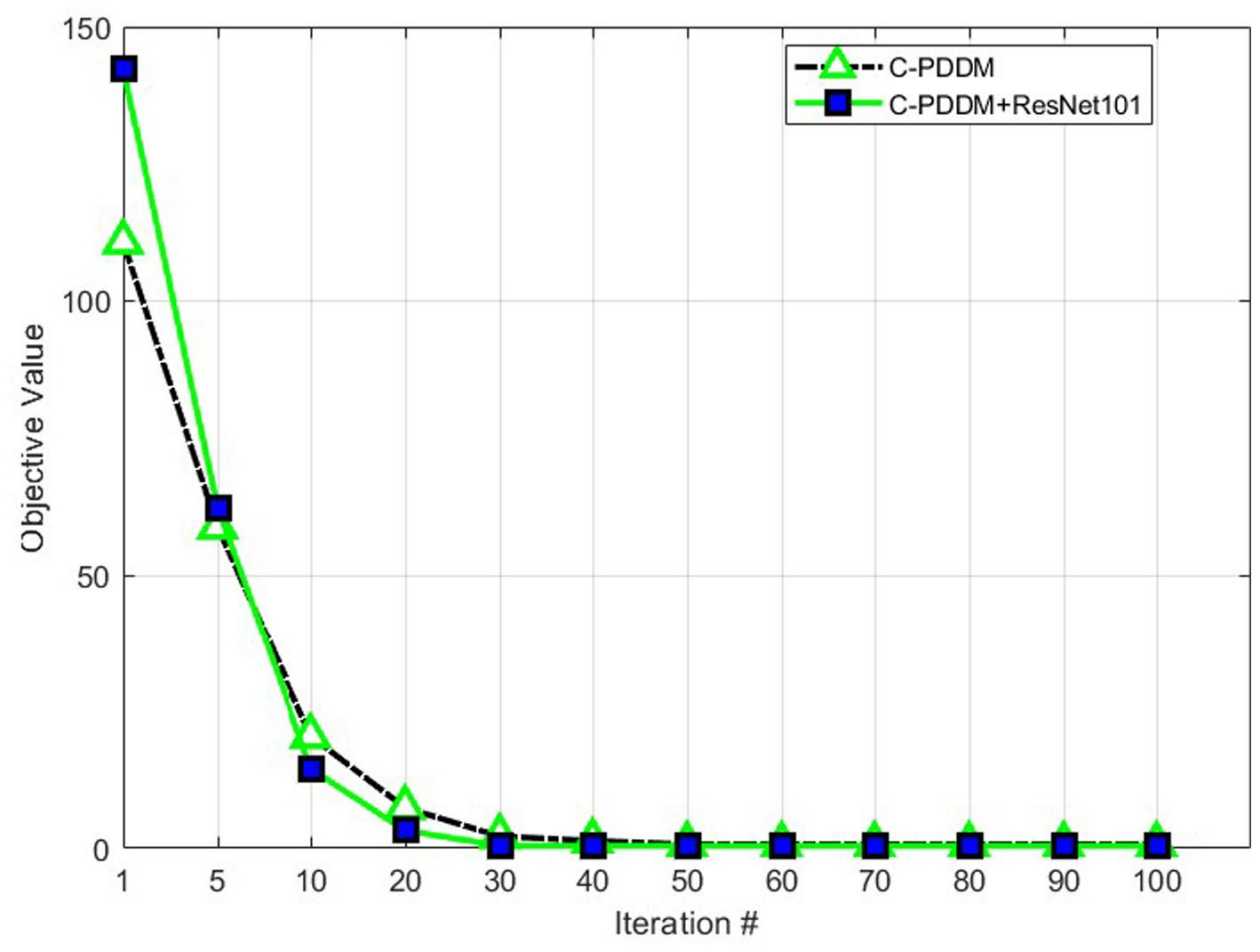
Convergence.

The proposed C-PDDM adopts an iterative optimization strategy and uses experiments to prove its convergence. The experiment is completed on the MATLAB platform, and the device configuration used is as follows: 64 GB memory, 2.5 GHz CPU, and 8-core Intel i7-11850H processor. Figure 4 shows the convergence process of C-PDDM at different iteration times. The results are shown in Figure 4. We can observe clearly that the proposed algorithm can achieve the minimum convergence at about 30 iterations. In the algorithm, the objective function of optimizing the sub-problem at each time is a decreasing function, which proves that the C-PDDM method has good convergence.

## 7. Conclusion

This paper proposes a novel transfer learning framework based on a Clustering-based Probability Distribution Distance Metric (C-PDDM) hypothesis, which uses a probability distribution distance metric criterion and fuzzy entropy technology for EEG data distribution alignment, and introduces the Laplace matrix to preserve the local structural information of source and target domain data. We evaluate the proposed C-PDDM model on two famous emotion databases (SEED and SEED-IV) and compare it with existing state-of-the-art methods under four cross-validation protocols (cross-subject single-session, single-subject single-session, single-subject cross-session, and cross-subject cross-session). Our extensive experimental results show that C-PDDM achieves the best results in most of the four cross-validation protocols, demonstrating the advantages of C-PDDM in dealing with individual differences and noisy label issues in aBCI systems.

## Data availability statement

The original contributions presented in the study are included in the article/supplementary material, further inquiries can be directed to the corresponding author.

## Author contributions

All authors listed have made a substantial, direct, and intellectual contribution to the work and approved it for publication.

## References

[ref1] Baktashmotlagh M. Harandi M. T. Lovell B. C. Salzmann M. (2013). “Unsupervised domain adaptation by domain invariant projection”. In Proc. the 2013 IEEE International Conference on Computer Vision, 769–776.

[ref2] Ben-David S. Blitzer J. Crammer K. Kulesza A. Pereira F. Vaughan J. W. (2010). A theory of learning from different domains. Mach. Learn. 79, 151–175. doi: 10.1007/s10994-009-5152-4

[ref3] Breiman (2001). Random forests. Mach. Learn. 45, 5–32. doi: 10.1023/A:1010933404324, PMID: 37872593

[ref4] Bruzzone L. Marconcini M. (2010). Domain adaptation problems: a DASVM classification technique and a circular validation strategy. IEEE Trans. Pattern Anal. Mach. Intell. 32, 770–787. doi: 10.1109/TPAMI.2009.57, PMID: 20299704

[ref5] Carlucci F. M Porzi L Caputo B Ricci E. (2017). Autodial: Automatic domain alignment layers. In: Proceeding of 2017 IEEE international conference on computer vision (ICCV), Venice, pp: 5077–5085.

[ref6] Chen H. Li Z. Jin M. Li J. (2021). “Meernet: multi-source EEG-based emotion recognition network for generalization across subjects and sessions” in 43rd annual international conference of the IEEE engineering in Medicine & Biology Society (EMBC), vol. 2021 (IEEE), 6094–6097. 10.1109/EMBC46164.2021.9630277 34892507

[ref7] Chen Z L Zhang J Y Liang X D Lin L. Blending-target domain adaptation by adversarial meta-adaptation networks. In: Proceeding of 2019 IEEE/CVF conference on computer vision and pattern recognition (CVPR), June 15-20, Long Beach (2019).

[ref8] Chu W.-S. Torre F. D. L. Cohn J. F. (2013). “Selective transfer machine for personalized facial action unit detection” in Proceeding of 2013 IEEE/CVF conference on computer vision and pattern recognition (CVPR) (Portland, OR), 3515–3522. 10.1109/CVPR.2013.451 PMC4169220 25242877

[ref9] Coomans D. Massart L. D. (1982). Alternative k-nearest neighbour rules in supervised pattern recognition: part 1. K-nearest neighbour classification by using alternative voting rules. Anal. Chim. Acta 136, 15–27. doi: 10.1016/S0003-2670(01)95359-0

[ref10] Dan Y. Tao J. Fu J. Zhou D. (2021). Possibilistic clustering-promoting semi-supervised learning for EEG-based emotion recognition. Front. Neurosci. 15:690044. doi: 10.3389/fnins.2021.690044, PMID: 34276295 PMC8281971

[ref11] Dan Y. Tao J. Zhou D. (2022). Multi-model adaptation learning with possibilistic clustering assumption for EEG-based emotion recognition. Front. Neurosci.:16. doi: 10.3389/fnins.2022(16):855421 PMC9114636 35600616

[ref12] Ding Z. M. Li S. Shao M. Fu Y. (2018). “Graph adaptive knowledge transfer for unsupervised domain adaptation” in European Proceeding of conference on computer vision (Munich), 36–52.

[ref13] Dolan R. J. (2002). Emotion, cognition, and behavior. Science 298, 1191–1194. doi: 10.1126/science.1076358, PMID: 12424363

[ref14] Du X. Ma C. Zhang G. Li J. Lai Y. K. Zhao G.. (2020). An efficient LSTM network for emotion recognition from multichannel EEG signals. IEEE Trans. Affect. Comput.:1. doi: 10.1109/TAFFC.2020.3013711

[ref15] Ganin Y. Ustinova E. Ajakan H. Germain P. Larochelle H. Laviolette F.. (2016). Domain-adversarial training of neural networks. J. Mach. Learn. Res. 17, 2096–2030. doi: 10.48550/arXiv.1505.07818

[ref16] Ghifary M. Balduzzi D. Kleijn W. B. Zhang M. (2017). Scatter component analysis: a unified framework for domain adaptation and domain generalization. IEEE Trans. Patt. Anal. Mach. Intell. 99:1. doi: 10.48550/arXiv.1510.04373 28113617

[ref17] Gong B. Shi Y. Sha F. Grauman K. (2012). Geodesic flow kernel for unsupervised domain adaptation. IEEE Conf. Comput. Vis. Patt. Recogn. 2012, 2066–2073. doi: 10.1109/CVPR.2012.6247911

[ref18] Gretton A. Borgwardt K. M. Rasch M. Scholkopf B. Smola A. J. (2007). “A kernel method for the two-sample-problem” in Proceeding of the 21st annual conference on neural information processing systems, December 3-6 (Vancouver, BC).

[ref19] Gretton A Harchaoui Z Fukumizu K J Harchaoui Z Sriperumbudur BK (2010). A fast, consistent kernel two-sample test. In: Proceedings of the 22nd international conference on neural information processing systems. 673–681. (Vancouver, BC, Canada).

[ref20] Jayaram V. Alamgir M. Altun Y. Scholkopf B. Grosse-Wentrup M. (2016). Transfer learning in brain-computer interfaces abstract. The performance of brain-computer interfaces (BCIs) improves with the amount of avail. IEEE Comput. Intell. Mag. 11, 20–31. doi: 10.1109/MCI.2015.2501545, PMID: 37857827

[ref21] Jenke R. Peer A. Buss M. (2014). Feature extraction and selection for emotion recognition from EEG. IEEE Trans. Affect. Comput. 5, 327–339. doi: 10.1109/TAFFC.2014.2339834, PMID: 37688466

[ref22] Jin J. Xiao R. Daly I. Miao Y. Wang X. Cichocki A. (2021). Internal feature selection method of CSP based on L1-norm and dempster–Shafer theory. IEEE Trans. Neural Netw. Learn. Syst. 32, 4814–4825. doi: 10.1109/TNNLS.2020.3015505, PMID: 32833646

[ref23] Kang G. L. Jiang L. Wei Y. Yang Y. Hauptmann A. (2022). Contrastive adaptation network for single- and multi-source domain adaptation. Inst. Elect. Electron. Eng. Trans. Patt. Anal. Mach. Intell. 44, 1793–1804. doi: 10.1109/TPAMI.2020.3029948, PMID: 33035160

[ref24] Kim M.-K. Kim M. Oh E. Kim S.-P. (2013). A review on the computational methods for emotional state estimation from the human EEG. Comput. Math. Methods Med. 2013:573734. doi: 10.1155/2013/573734, PMID: 23634176 PMC3619694

[ref25] Krishnapuram R. Keller J.-M. (1993). A possibilistic approach to clustering. IEEE Trans. Fuzzy Syst. 1, 98–110. doi: 10.1109/91.227387, PMID: 36262121

[ref26] Lan Z. Sourina O. Wang L. Scherer R. Muller-Putz G. R. (2019). Domain adaptation techniques for EEG-based emotion recognition: a comparative study on two public datasets. IEEE Trans. Cogn. Dev. Syst. 11, 85–94. doi: 10.1109/TCDS.2018.2826840

[ref27] Lee S M Kim D W Kim N Jeong SG. Drop to adapt: Learning discriminative features for unsupervised domain adaptation. In: Proceeding of 2019 IEEE/CVF international conference on computer vision (ICCV), October 27-November 2, Seoul (2019). pp: 90–100.

[ref28] Li J. Qiu S. du C. Wang Y. He H. (2020). Domain adaptation for EEG emotion recognition based on latent representation similarity. IEEE Trans. Cogn. Dev. Syst. 12, 344–353. doi: 10.1109/TCDS.2019.2949306

[ref29] Li H. Jin Y. M. Zheng W. L. Lu B. L. (2018d). “Cross-subject emotion recognition using deep adaptation networks” in Neural information processing. eds. Cheng L. Leung A. C. S. Ozawa S. (Cham: Springer International Publishing), 403–413.

[ref30] Li X. Song D. Zhang P. Zhang Y. Hou Y. Hu B. (2018). Exploring EEG features in cross-subject emotion recognition. Front. Neurosci. 12:162. doi: 10.3389/fnins.2018.00162, PMID: 29615853 PMC5867345

[ref31] Li Y. Wang L. Zheng W. Zong Y. Qi L. Cui Z.. (2020). A novel bi-hemispheric discrepancy model for EEG emotion recognition. IEEE Trans. Cogn. Dev. Syst. 13, 354–367. doi: 10.1109/TCDS.2020.2999337

[ref32] Li Y. Zheng W. Cui Z. Zhang T. Zong Y. A novel neural network model based on cerebral hemispheric asymmetry for EEG emotion recognition. The 27th international joint conference on artificial intelligence (IJCAI) (2018b).

[ref33] Li Y. Zheng W. Cui Z. Zong Y. Ge S. (2018a). EEG emotion recognition based on graph regularized sparse linear regression. Neural. Process. Lett. 49, 555–571. doi: 10.1007/s11063-018-9829-1

[ref34] Li Y. Zheng W. Wang L. Zong Y. Cui Z. (2019). From regional to global brain: a novel hierarchical spatial-temporal neural network model for EEG emotion recognition. IEEE Trans. Affect. Comput. doi: 10.1109/TAFFC.2019.2922912

[ref35] Li Y. Zheng W. Zong Y. Cui Z. Zhang T. Zhou X. (2018c). A bi-hemisphere domain adversarial neural network model for EEG emotion recognition. IEEE Trans. Affect. Comput. 12, 494–504. doi: 10.1109/TAFFC.2018.2885474

[ref36] Liang J. He R. Sun Z. N. Tan T. (2018). Aggregating randomized clustering-promoting invariant projections for domain adaptation. Inst. Electr. Electron. Eng. Trans. Patt. Anal. Mach. Intell. 41, 1027–1042. doi: 10.1109/TPAMI.2018.2832198, PMID: 29993436

[ref37] Long M. Cao Y. Wang J. Jordan M., Learning transferable features with deep adaptation networks. In: Proceedings of the 32nd international conference on international conference on machine learning, Lille, 97–105 (2015).

[ref38] Long M S Wang J M Ding G G Sun J Yu PS. Transfer feature learning with joint distribution adaptation. In: Proceedings of the 2013 IEEE international conference on computer vision. IEEE, (2013).

[ref39] Long M. S. Wang J. M. Jordan M. I. (2016). “Unsupervised domain adaptation with residual transfer networks” in Proceeding of the 30th Annual conference on neural information processing systems, December 5-10 (Barcelona), 136–144.

[ref40] Luo L. K. Chen L. M. Hu S. Q. Lu Y. Wang X. (2020). Discriminative and geometry aware unsupervised domain adaptation. IEEE Trans. Cybern. 50, 3914–3927. doi: 10.1109/TCYB.2019.2962000, PMID: 31976922

[ref41] Luo Y. Zhang S. Y. Zheng W. L. Lu BL. Wgan domain adaptation for EEG-based emotion recognition, In: International Conference on Neural Information Processing (2018).

[ref42] Ma B.-Q. Li H. Zheng W.-L. Lu B.-L. (2019). “Reducing the subject variability of eeg signals with adversarial domain generalization” in Neural information processing. eds. Gedeon T. Wong K. W. Lee M. (Cham: Springer International Publishing), 30–42.

[ref43] Mühl C. Allison B. Nijholt A. Chanel G. (2014). A survey of affective brain computer interfaces: principles, state-of-the-art, and challenges. Brain Comput. Interfaces 1, 66–84. doi: 10.1080/2326263X.2014.912881

[ref44] Musha T. Terasaki Y. Haque H. A. Ivamitsky G. A. (1997). Feature extraction from EEGs associated with emotions. Artif. Life Robot. 1, 15–19. doi: 10.1007/BF02471106

[ref45] Nie F P Huang H Cai X Huang H. Efficient and robust feature selection via joint -norms minimization. In: Proceedings of the 23rd international conference on neural information processing systems. Curran Associates Inc (2010): 1813–1821.

[ref46] Pan S. J. Tsang I. W. Kwok J. T. Yang Q. (2011). Domain adaptation via transfer component analysis. IEEE Trans. Neural Netw. 22, 199–210. doi: 10.1109/TNN.2010.2091281, PMID: 21095864

[ref47] Pan S. J. Yang Q. (2010). A survey on transfer learning. IEEE Trans. Knowl. Data Eng. 22, 1345–1359. doi: 10.1109/TKDE.2009.191, PMID: 37871059

[ref48] Pandey P. Seeja K. “Emotional state recognition with EEG signals using subject independent approach” Lecture notes on data engineering and communications technologies, data science and big data analytics, (Springer) (2019) 117–124. doi: 10.1.7/978-981-10-7641-1_10

[ref49] Patel V. M. Gopalan R. Li R. Chellappa R. (2015). Visual domain adaptation: a survey of recent advances. IEEE Signal Process. Mag. 32, 53–69. doi: 10.1109/MSP.2014.2347059, PMID: 37030853

[ref50] Pinheiro P. O. (2018). Unsupervised domain adaptation with similarity learning. IEEE/CVF Conf. Comput. Vis. Patt. Recogn. 2018, 8004–8013. doi: 10.48550/arXiv.1711.08995

[ref51] Shi L.-C. Lu B.-L. (2010). Off-line and on-line vigilance estimation based on linear dynamical system and manifold learning. Annu. Int. Conf. IEEE Eng. Med. Biol. 2010, 6587–6590. doi: 10.1109/IEMBS.2010.5627125, PMID: 21096513

[ref52] Song T. Zheng W. Song P. Cui Z. (2018). EEG emotion recognition using dynamical graph convolutional neural networks. IEEE Trans. Affect. Comput. 11:1. doi: 10.1109/BIBM.2018.8621147

[ref53] Sriperumbudur B K Fukumizu K Gretton A GRG Lanckriet Scholkopf B. Kernel choice and classifiability for RKHS embeddings of probability distributions. In: Proceeding of the 23rd annual conference on neural information processing systems (NIPS 2009). Red Hook, NY: MIT Press, 2010:1750–1758 (2010a).

[ref54] Sriperumbudur B. K. Gretton A. Fukumizu K. GRG L. Scholkopf B. (2010b). Hilbert space embeddings and metrics on probability measures. J. Mach. Learn. Res. 11, 1517–1561. doi: 10.1007/s10846-009-9337-7

[ref55] Sun B. Feng J. Saenko K., Return of frustratingly easy domain adaptation. In: Proceedings of the thirtieth AAAI conference on artificial intelligence, ser. AAAI’16. AAAI Press, (2016), p. 2058–2065.

[ref56] Sun Y. Gao Y. Zhao Y. Liu Z. Wang J. Kuang J.. (2022). Neural network-based tracking control of uncertain robotic systems: predefined-time nonsingular terminal sliding-mode approach. IEEE Trans. Ind. Electron. 69, 10510–10520. doi: 10.1109/TIE.2022.3161810

[ref58] Suykens J. Vandewalle J. (1999). Least squares support vector machine classifiers. Neural. Process. Lett. 9, 293–300. doi: 10.1023/A:1018628609742, PMID: 37835246

[ref59] Tang H Jia K, Discriminative adversarial domain adaptation. In: Proceeding of the 34th National Conference on artificial intelligence, Feb. 7-12, New York (2019).

[ref60] Tao J. Chung F. L. Wang S. (2012). On minimum distribution discrepancy support vector machine for domain adaptation. Pattern Recogn. 45, 3962–3984. doi: 10.1016/j.patcog.2012.04.014

[ref61] Tao J. W. Dan Y. F. (2021). Multi-source co-adaptation for EEG-based emotion recognition by mining correlation information. Front. Neurosci. 15:677106. doi: 10.3389/fnins.2021.677106, PMID: 34054422 PMC8155359

[ref62] Tao J. Dan Y. Di Z. (2021). Robust multi-source co-adaptation with adaptive loss minimization. Signal Process. Image Commun. 99:116455. doi: 10.1016/j.image.2021.116455

[ref63] Tao J. Dan Y. F. Zhou D. He S. S. (2022). Robust latent multi-source adaptation for encephalogram-based emotion recognition. Front. Neurosci. 16:850906. doi: 10.3389/fnins.2022.850906, PMID: 35573289 PMC9091911

[ref64] Tao J. Di Zhou F. L. Zhu B. (2019). Latent multi-feature co-regression for visual recognition by discriminatively leveraging multi-source models. Pattern Recogn. 87, 296–316. doi: 10.1016/j.patcog.2018.10.023

[ref65] Tao J. W. Song D. Wen S. Hu W. (2017). Robust multi-source adaptation visual classification using supervised low-rank representation. Pattern Recogn. 61, 47–65. doi: 10.1016/j.patcog.2016.07.006

[ref66] Tao J. Wen S. Hu W. (2015). L1-norm locally linear representation regularization multi-source adaptation learning. Neural Netw. 69, 80–98. doi: 10.1016/j.neunet.2015.01.009, PMID: 26091754

[ref67] Tao J. Wen S. Hu W. (2016). Multi-source adaptation learning with global and local regularization by exploiting joint kernel sparse representation. Knowl. Based Syst. 98, 76–94. doi: 10.1016/j.knosys.2016.01.021

[ref68] Tzeng E. Hoffman J. Zhang N. Saenko K. Darrell T. (2014). Deep domain confusion: maximizing for domain invariance. CoRR abs/1412.3474 Available at: http://arxiv.org/abs/1412.3474

[ref69] Wang J. Ji Z. Kim H. E. Wang S. Xiong L. Jiang X. (2017). Selecting optimal subset to release under differentially private M-estimators from hybrid datasets. IEEE Trans. Knowl. Data Eng. 30, 573–584. doi: 10.1109/TKDE.2017.2773545, PMID: 30034201 PMC6051552

[ref70] Xiao X. Xu M. Jin J. Wang Y. Jung T. P. Ming D. (2020). Discriminative canonical pattern matching for single-trial classification of erp components. IEEE Trans. Biomed. Eng. 67, 2266–2275. doi: 10.1109/TBME.2019.2958641, PMID: 31831401

[ref71] Zhang Y. Dong J. Zhu J. Wu C. (2019b). Common and special knowledge-driven TSK fuzzy system and its modeling and application for epileptic EEG signals recognition. IEEE Access, 2019 7, 127600–127614. doi: 10.1109/ACCESS.2019.2937657

[ref72] Zhang Y. Tian F. Wu H. Geng X. Qian D. Dong J.. (2017). Brain MRI tissue classification based fuzzy clustering with competitive learning. J. Med. Imaging Health Informat. 7, 1654–1659. doi: 10.1166/jmihi.2017.2181, PMID: 8812077

[ref73] Zhang Y. Wang S. Xia K. Jiang Y. Qian P. (2021). Alzheimer’s disease multiclass diagnosis via multimodal neuroimaging embedding feature selection and fusion. Informat. Fusion 66, 170–183. doi: 10.1016/j.inffus.2020.09.002

[ref74] Zhang T. Zheng W. Cui Z. Zong Y. Li Y. (2019a). Spatial–temporal recurrent neural network for emotion recognition. IEEE Trans. Cybern. 49, 839–847. doi: 10.1109/TCYB.2017.2788081 29994572

[ref75] Zheng W. (2017). Multichannel EEG-based emotion recognition via group sparse canonical correlation analysis. IEEE Trans. Cogn. Dev. Syst. 9, 281–290. doi: 10.1109/TCDS.2016.2587290

[ref76] Zheng W.-L. Liu W. Lu Y. Lu B. L. Cichocki A. (2019). EmotionMeter: a multimodal framework for recognizing human emotions. IEEE Trans. Cybern. 49, 1110–1122. doi: 10.1109/TCYB.2018.2797176, PMID: 29994384

[ref77] Zheng W.-L. Lu B.-L. (2015). Investigating critical frequency bands and channels for EEG-based emotion recognition with deep neural networks. IEEE Trans. Auton. Ment. Dev. 7, 162–175. doi: 10.1109/TAMD.2015.2431497

[ref78] Zheng W. L. Lu B. L. Personalizing EEG-based affective models with transfer learning. Proceedings of the Twenty-Fifth International Joint Conference on Artificial Intelligence, AAAI Press (2016), pp. 2732–2738.

[ref79] Zheng W. L. Zhang Y. Q. Zhu J. Y. Lu B. L. (2015). “Transfer components between subjects for EEG-based emotion recognition” in International conference on affective computing and intelligent interaction (ACII) (Xi'an), 917–922.

[ref80] Zhong P. Wang D. Miao C. (2020). EEG-based emotion recognition using regularized graph neural networks. IEEE Trans. Affect. Comput. doi: 10.48550/arXiv.1907.07835

[ref81] Zhou R. Zhang Z. Fu H. Zhang L. Li L. Huang G.. (2022). A novel transfer learning framework with prototypical representation based pairwise learning for cross-subject cross-session EEG-based emotion recognition. ArXiv abs/2202.06509. doi: 10.48550/arXiv.2202.06509

[ref82] Zhu J. Arbor A. Hastie T. (2006). Multi-class adaboost. Stat. Interface 2, 349–360. doi: 10.4310/SII.2009.v2.n3.a8

